# Super broad and protective nanobodies against Sarbecoviruses including SARS-CoV-1 and the divergent SARS-CoV-2 subvariant KP.3.1.1

**DOI:** 10.1371/journal.ppat.1012625

**Published:** 2024-11-11

**Authors:** Haodi Dong, Runhong Zhou, Jing Chen, Jing Wei, Zimeng Wei, Ziqing Yang, Kun Zhu, Yufan Yang, Qianqian Yang, Na Liu, Yuting Chen, Yuhan Wu, Yan Liang, Yige Zeng, Qile Guo, Mingxi Li, Sisi Shan, Han Wang, Mengyue Niu, Isabella Yunfei Zeng, Xuanling Shi, Qi Zhang, Xinquan Wang, Zhiwei Chen, Linqi Zhang

**Affiliations:** 1 Comprehensive AIDS Research Center, Pandemic Research Alliance Unit, Center for Infection Biology, School of Basic Medical Sciences, Tsinghua Medicine, Tsinghua University, Beijing, China; 2 AIDS Institute, School of Clinical Medicine, Li Ka Shing Faculty of Medicine, The University of Hong Kong, Pokfulam, Hong Kong Special Administrative Region, People’s Republic of China; 3 Department of Microbiology, School of Clinical Medicine, Li Ka Shing Faculty of Medicine, The University of Hong Kong, Pokfulam, Hong Kong Special Administrative Region, People’s Republic of China; 4 Centre for Virology, Vaccinology and Therapeutics, Hong Kong Science and Technology Park, Hong Kong Special Administrative Region, People’s Republic of China; 5 The Ministry of Education Key Laboratory of Protein Science, Beijing Advanced Innovation Center for Structural Biology, Beijing Frontier Research Center for Biological Structure, Collaborative Innovation Center for Biotherapy, School of Life Sciences, Tsinghua University, Beijing, China; 6 School of Engineering, Massachusetts Institute of Technology, Cambridge, Massachusetts, United States; 7 Institute of Biopharmaceutical and Health Engineering, Tsinghua Shenzhen International Graduate School, Tsinghua University, Shenzhen, China; 8 Institute of Biomedical Health Technology and Engineering, Shenzhen Bay Laboratory, Shenzhen, China; Institut Pasteur, FRANCE

## Abstract

The ongoing evolution and immune escape of SARS-CoV-2, alongside the potential threat of SARS-CoV-1 and other sarbecoviruses, underscore the urgent need for effective strategies against their infection and transmission. This study highlights the discovery of nanobodies from immunized alpacas, which demonstrate exceptionally broad and potent neutralizing capabilities against the recently emerged and more divergent SARS-CoV-2 Omicron subvariants including JD.1.1, JN.1, KP.3, KP.3.1.1, as well as SARS-CoV-1 and coronaviruses from bats and pangolins utilizing receptor ACE2. Among these, Tnb04-1 emerges as the most broad and potent, binding to a conserved hydrophobic pocket in the spike’s receptor-binding domain, distinct from the ACE2 binding site. This interaction disrupts the formation of a proteinase K-resistant core, crucial for viral-cell fusion. Notably, intranasal administration of Tnb04-1 in Syrian hamsters effectively prevented respiratory infection and transmission of the authentic Omicron XBB.1.5 subvariant. Thus, Thb04-1 holds promise in combating respiratory acquisition and transmission of diverse sarbecoviruses.

## Author summary

As SARS-CoV-2 evolves and evades antibody immunity, new antibody intervention strategies are urgently needed to prevent viral acquisition and transmission. We identified a cluster of nanobodies from immunized alpacas with broad and potent neutralizing activity against all major SARS-CoV-2 variants, SARS-CoV-1, and ACE2-utilizing coronaviruses from bats and pangolins. Tnb04-1 was the most effective, binding to a conserved epitope in spike and providing strong protection against contact and respiratory infection of Omicron XBB.1.5 in Syrian hamsters. Tnb04-1 shows promise for next-generation antibody interventions against diverse sarbecoviruses.

## Introduction

On May 5, 2023, after over three years of extensive global efforts, the World Health Organization declared the end of the COVID-19 Public Health Emergency [1, 2]. While this marks a significant milestone, it does not indicate the eradication of SARS-CoV-2, nor does it imply the complete resolution of the pandemic’s impacts. To the contrary, there has been a recent global surge in outbreaks, resulting in increased COVID-19 hospitalizations and associated spread of new Omicron subvariants [3]. Notably, while the EG.5 subvariant, an XBB derivative, is prevalent in numerous regions, the BA.2.86 descendant, JN.1, is poised to become the next dominant variant worldwide. In addition, ample evidence had already demonstrated that EG.5 and JN.1 can evade antibody immunity in vaccinated and/or infected individuals [4, 5]. Additionally, with the emergence of the KP.3 and KP.3.1.1 variants now dominant in many regions worldwide, another wave of antibody evasion has been reported [6–8]. This dynamic pattern aligns closely with the trajectory of previous variants, which emerged, dominated, and were eventually supplanted by more divergent and transmissible successors. Consequently, the cycle of immune selection and viral escape is expected to persist, posing ongoing challenges to antibody drugs and vaccines [9, 10]. Moreover, the potential threat of other sarbecoviruses, such as SARS-CoV-1, emphasizes the urgent need for next-generation antibodies and vaccines capable of disrupting the cycle of immune selection and viral escaping, preferably by blocking respiratory infection and transmission of diverse sarbecovirus strains.

Since the initial outbreak of the COVID-19 pandemic, both our team and other researchers have concentrated on analyzing antibody responses in infected humans and immunized animals [9–26]. Through single-B-cell and antibody-library based strategies, a large number of human monoclonal antibodies (mAbs) and nanobodies (nbs) have been isolated and characterized for their structure and function properties [9–30]. The common theme emerging from these studies is that the receptor-binding domain (RBD) of the viral spike serves as the primary target for neutralizing antibodies (nAb), succeeded by the N-terminal domain (NTD), the fusion peptide, and the interface between the NTD and subdomain 1 (SD1)[9, 10]. A substantial fraction of mAbs targeting the S2 portion of the spike has also been identified [16]. However, they seldom exhibit neutralizing capability in vitro although some have conferred protection in animal models [31]. As SARS-CoV-2 continues to evolve, particularly when Omicron subvariants appeared and spread globally, the once broadly effective nAbs have severely been compromised [9, 10, 32–38]. Furthermore, the recently emerged Omicron subvariants EG.5, HK3, HV.1, JD.1.1, BA.2.86, JN.1, KP.3, and KP.3.1.1 possess an even higher number of spike substitutions compared to earlier subvariants like BA.1, BA.2, and BA.4/5. This increase has led to further evasion from antibody responses in both infected humans and immunized animals [6–8, 36–38]. Identifying antibodies that can counter these diverse SARS-CoV-2 variants remains a formidable challenge.

Here, we report on the identification of a set of nanobodies with extremely broad and potent neutralizing activity against diverse human and animal sarbecoviruses, including the recently emerged Omicron subvariants EG.5, HK3, HV.1, JD.1.1, BA.2.86, JN.1, KP.3, and KP.3.1.1 as well as SARS-CoV-1 and coronaviruses from bats and pangolins that utilize the receptor ACE2. Tnb04-1, the best among these nanobodies, recognized a highly conserved and unique epitope comprising a hydrophobic pocket in the RBD, distinct from the ACE2 receptor binding site. Mechanistically, Tnb04-1 appears to interfere the formation of a proteinase K-resistant core, essential for the transition of the spike trimer from a pre-hairpin intermediate to a six-helix bundle essential for viral-cell fusion. When administrated intranasally, Tnb04-1 demonstrated robust protection in Syrian hamsters against both contact and respiratory transmission of the authentic Omicron XBB.1.5 subvariant. Thus, Tnb04-1 emerges as a promising candidate for disrupting the cycle of immune selection and viral escape through blocking respiratory acquisition and transmission of diverse sarbecoviruses.

## Results

### Identification of broadly neutralizing nanobodies against SARS-CoV-1, SARS-CoV-2 and sarbecoviruses

**Fig 1:**
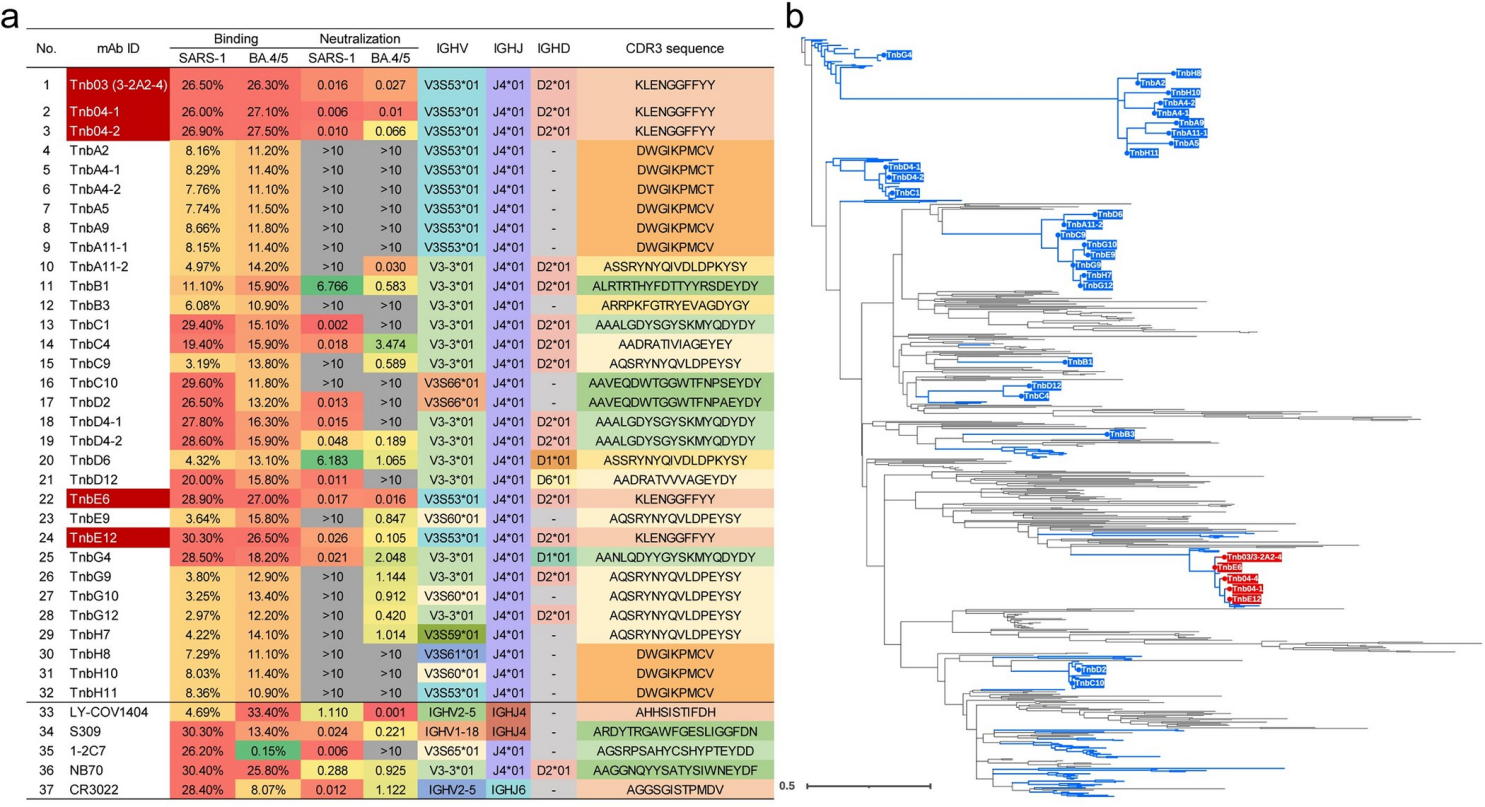
Binding, neutralizing, germline and phylogenetic properties of isolated nanobodies against SARS-CoV-1 and SARS-CoV-2 BA.4/5 variant. **a.** Binding activity is indicated by the percent (%) of spike positive cells analyzed by flow cytometry with red being the highest, followed by orange, and green. Neutralizing activity is shown by IC50 (μg/mL) and colored in red, orange, yellow, green, and gray, with red being the strongest and gray failed to reach IC50 at the highest concentration tested (10μg/ml). The five nanobodies highlighted in red in the column of mAb ID are the ones selected for downstream evaluation. All results were calculated from at least two independent experiments. The germline variable gene segment (V), diversity gene segment (D), and junction gene (J) as well as CDR3 sequences of each nanobody are colored for clarity. **b.** Phylogenetic analysis of nanobodies isolated in this study indicated by their names at the tip of the branches, compared with those isolated previously by our group (blue line) [28] and randomly selected from CoV-AbDab database (https://opig.stats.ox.ac.uk/webapps/covabdab/) (black line).

Since our early report on the isolation of few nanobodies against diverse human and animal coronaviruses [28], numerous more divergent Omicron subvariants have emerged and spread worldwide. These include BF7, BQ.1, BQ.1.1, XBB.1.5, XBB.1.16, CH.1.1, and notably the recent EG.5, HK3, HV.1, JD.1.1, BA.2.86, JN.1, KP.3, and KP.3.1.1 [6, 7, 37, 39–41]. To identify nanobodies with enhanced breadth and potency, we refined our selection protocol using spikes from SARS-CoV-1 and SARS-CoV-2 Omicron subvariant BA.4/5 as baits, with the premise that nanobodies reacting positively to both spikes are likely to cross-react with new Omicron subvariants and other sarbecoviruses. Through iterative MACS-sorting, FACS-sorting, enrichment, and expression in recombinant form with human IgG1 Fc fragment, we isolated 32 VHH nanobodies binding to the spike trimer of SARS-CoV-1 and SARS-CoV-2 Omicron BA.4/5 (Fig 1A). Among these, ten exhibited cross-neutralizing activity against SARS-CoV-1 and BA.4/5 pseudoviruses, with IC50 values ranging from 0.001 to 6.766 μg/mL. The top five nanobodies–Tnb03 (3-2A2-4), Tnb04-01, Tnb04-02, TnbE6, and TnbE12 –were chosen for further evaluation alongside control antibodies (LY-COV1404, S309, 1-2C7, NB70, and CR3022) previously published (Fig 1A) [28, 42–44]. Genetically, these nanobodies shared germline genes IGHV3S53, IGHJ4, and D2*01 and the CDR3 sequence KLENGGFFYY, albeit with minor differences in other regions (S1 Fig). This germline and CDR3 convergence suggest that their unique structural and biochemical characteristics naturally complement the spike surface of SARS-CoV-1 and SARS-CoV-2 Omicron BA.4/5. Phylogenetic analysis revealed that these top nanobodies formed a distinct cluster (red) on the tree, distinct from other nanobodies isolated here and our previous studies (blue) and representative nanobodies from the CoV-AbDab database (black) (Fig 1B). Notably, Tnb03 was identical to previously isolated nanobody 3-2A2-4 (S1 Fig), which demonstrated extensive breadth and potency against 15 human and animal sarbecoviruses [28].

### Tnb04-1 displayed the highest neutralizing activity against a wide range of sarbecoviruses

**Fig 2:**
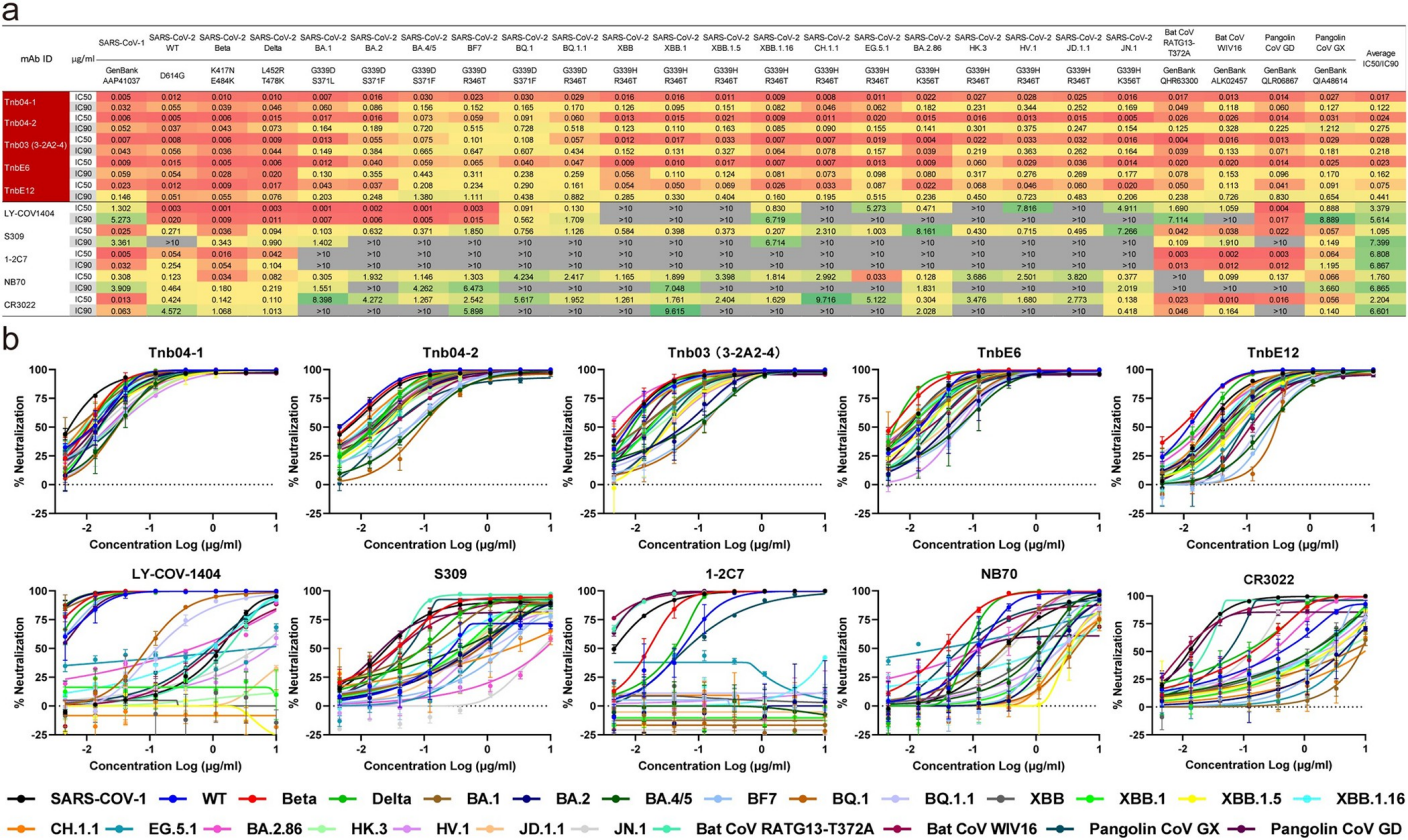
Neutralization breadth and potency of top 5 nanobodies against 25 diverse sarbecoviruses. **a.** Nanobody concentrations (μg/ml) required to achieve 50% (IC50) and 90% (IC90) reduction in viral infection. For clarity, neutralizing activity of each nanobody and control antibody is also colored with decreasing sequence from red, orange, yellow, green, to gray. Those in gray failed to reach IC50 at the highest concentration (10μg/ml) tested. A few representative escape mutations found in SARS-CoV-2 variants, and the GenBank accession numbers of SARS-CoV-1 and hACE2-dependent bat and pangolin coronaviruses are indicated under each strain tested. The complete set of mutations in each of the SARS-CoV-2 variant are indicated in the methods section. **b.** Actual neutralizing curve of nanobodies and control antibodies against the 25 diverse pseudoviruses, from which the IC50 and IC90 are estimated. The results shown are representatives of two independent experiments and presented as mean ± SEM.

We next tested neutralizing activity of the top five nanobodies against a panel of 25 pseudoviruses bearing the S protein from various sarbecoviruses, including prototype SARS-CoV-1, 14 SARS-CoV-2 prototypes and major subvariants, 6 recent Omicron subvariants EG.5, HK3, HV.1, JD.1.1, BA.2.86, and JN.1, and 4 receptor ACE2-using bat and pangolin coronaviruses (Fig 2). Tnb04-1 showed the most robust neutralization with an average IC50 of 0.017 μg/ml and IC90 of 0.122 μg/ml, outperforming Tnb04-2 (IC50: 0.024 μg/ml and IC90: 0.275 μg/ml), Tnb03 (3-2A2-4) (IC50: 0.028 μg/ml and IC90: 0.218 μg/ml), TnbE6 (IC50: 0.023 μg/ml and IC90: 0.162 μg/ml), and TnbE12 (IC50: 0.075 μg/ml and IC90: 0.441 μg/ml) (Fig 2A). Tnb04-1 not only maintained its high neutralizing efficacy against recent Omicron subvariants EG.5, HK3, HV.1, JD.1.1, BA.2.86, and JN.1 but also showed exceptional activity against SARS-CoV-1 (average IC50: 0.005 μg/ml) and bat and pangolin coronaviruses (average IC50: 0.018 μg/ml) (Fig 2A). The remaining four nanobodies showed moderate reduction in IC50 and IC90 particularly to the earlier Omicron subvariants such as BA.4/5, BF.7, and BQ.1 compared to the wildtype SARS-CoV-2 (Fig 2A). The largest reduction was found among the control antibodies (Fig 2). Additionally, during the revision of the manuscript, the KP.3 and KP.3.1.1 variants emerged and became dominant in many regions globally. We demonstrated that Tnb04-1 retained strong neutralizing activity against both variants, with an IC50 of 0.002 μg/ml and an IC90 of 0.356 μg/ml for KP.3, and an IC50 of 0.008 μg/ml and an IC90 of 0.273 μg/ml for KP.3.1.1 μg/ml (S4 Fig). These findings clearly establish Tnb04-1 as the most effective inhibitor across a broad range of human and animal sarbecoviruses.

### Tnb04-1 exhibits the strongest inhibitory activity against cell-cell fusion mediated by diverse SARS-CoV-2 spikes

**Fig 3:**
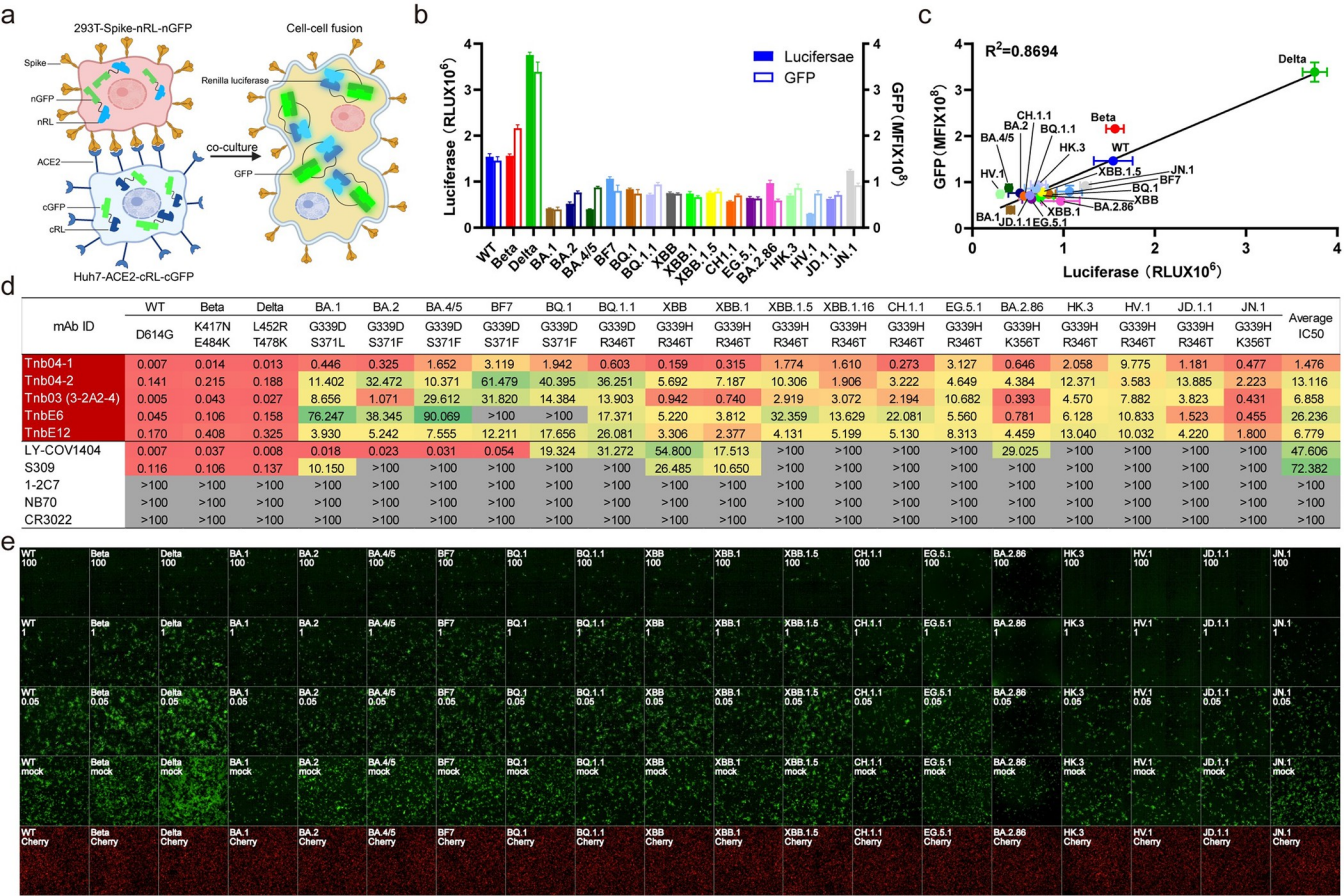
Inhibition of diverse spike-mediated cell-cell fusion by isolated nanobodies. **a.** Cartoon illustration of a dually-split protein (DSP) assay to quantitatively measure cell-cell fusion by luciferase and GFP activity between two reporter cell lines: 293T cells expressing spike and nRL-nGFP (N-*Renilla* Luciferase-GFP1-7) and Huh-7 cells expressing the endogenous ACE2 and cRL-cGFP (C-N-*Renilla* Luciferase-GFP8-11). The cartoon was created with BioRender.com. **b.** Comparison of fusion activity of diverse spike of SARS-CoV-2 variants. **c.** Correlation between luciferase- and GFP-measured fusion activity. **d.** The potency and breadth of the top 5 isolated nanobodies and control antibodies in inhibiting cell-cell fusion mediated by a panel of 20 spike variants. Numbers indicate the nanobody and antibody concentrations (μg/ml) required to achieve 50% (IC50) reduction in cell-cell fusion. For clarity, inhibitory activity of each nanobody and control antibody is colored with decreasing sequence from red, orange, yellow, green, to gray. Those in gray failed to reach IC50 at the highest concentration tested (100μg/ml). A few representative mutations that potentially facilitate viral escape from antibodies are indicated below each of variants tested. The complete set of mutations in each of the variant are indicated in the methods section. **e.** Inhibitory activity of the best nanobody Tnb04 on cell-cell fusion shown by fluorescence imaging, captured and measured by the Opera Phenix Plus High-Content Screening System (PerkinElmer). Fluorescence intensity against each variant was first normalized against internal control mCherry before comparison was performed. Three concentrations of nanobody Tnb04 (100μg/ml, 1μg/ml, 0.05μg/ml) were tested against each and every variant listed.

We used a dually-split protein (DSP) assay to investigate the fusion activity of the spikes of 20 SARS-CoV-2 variants, quantitatively measured by the relative light unit (RLU) of luciferase and mean fluorescent intensity (MFI) of GFP (Fig 3A) [45]. The wildtype (WT), Beta, and Delta mediated robust cell-cell fusion, with Delta being the strongest (RLU of 3.754), followed by Beta (RLU of 1.564), and then WT (RLU of 1.544) (Fig 3B). The Omicron variants showed substantially lower fusion activity measured by both RLU and MFI, although BF.7, BA.2.86 and JN.1 had minor improvement. Importantly, the fusion activity measured by RLU and MFI are highly correlated (R: 0.8694) (Fig 3C), validating the robustness of the DSP-based assay for measuring fusion of SARS-CoV-2 spikes.

Next, we evaluated the inhibitory activity of the top five nanobodies against spike-mediated cell-cell fusion together with control antibodies LY-COV1404, S309, 1-2C7, NB70, and CR3022. Consistent with pseudovirus neutralization, Tnb04-1 emerged as the most effective inhibitor, with an average IC50 of 1.476 μg/ml against spikes from WT, Beta, Delta, and diverse Omicron subvariants, including recently identified and highly divergent strains EG.5, BA.2.86, HK3, HV.1, JD.1.1, and JN.1 (Fig 3D). The Tnb04-2, Tnb03 (3-2A2-4), TnbE6, and TnbE12 showed relative weaker activity, particularly to early Omicron subvariants such as BA.1, BA.2, BA.4/5, BF.7, BQ.1, and BQ.1.1. The control antibodies exhibited significant lower activity and failed to inhibit many spike-mediated fusion especially to the most recent HK3, HV.1, JD.1.1, and JN.1 (Fig 3D). Notably, the IC50 values against cell-cell fusion were approximately 100- to 1000-fold weaker than those against pseudoviruses (Figs 2 and S2). In many instances, the inhibitory activity failed to reach 100% (S3 Fig), likely due to higher spike and ACE2 expression on effector and target cells that require substantially higher nanobody concentrations for complete inhibition.

We next evaluated anti-fusion activity of Tnb04-1 by measuring GFP-positive cells captured by high-content screening systems (Fig 3E). A clear dose-effect of Tnb04-1 was found against each spike-mediated fusion as more GPF-positive cells became detectable as the concentration of Tnb04-1 decreased from 100 to 0.05μg/ml. In the mock group, clear differences in fusion efficiency were found among different spikes tested (Fig 3E and 3B). Collectively, these results show that Tnb04-1 exhibits the strongest activity not only to spike-mediated entry of pseudovirus but also spike-mediated cell-cell fusion.

### Tnb04-1 neutralizes a range of authentic SARS-CoV-2 variants and SARS-CoV-1

**Fig 4:**
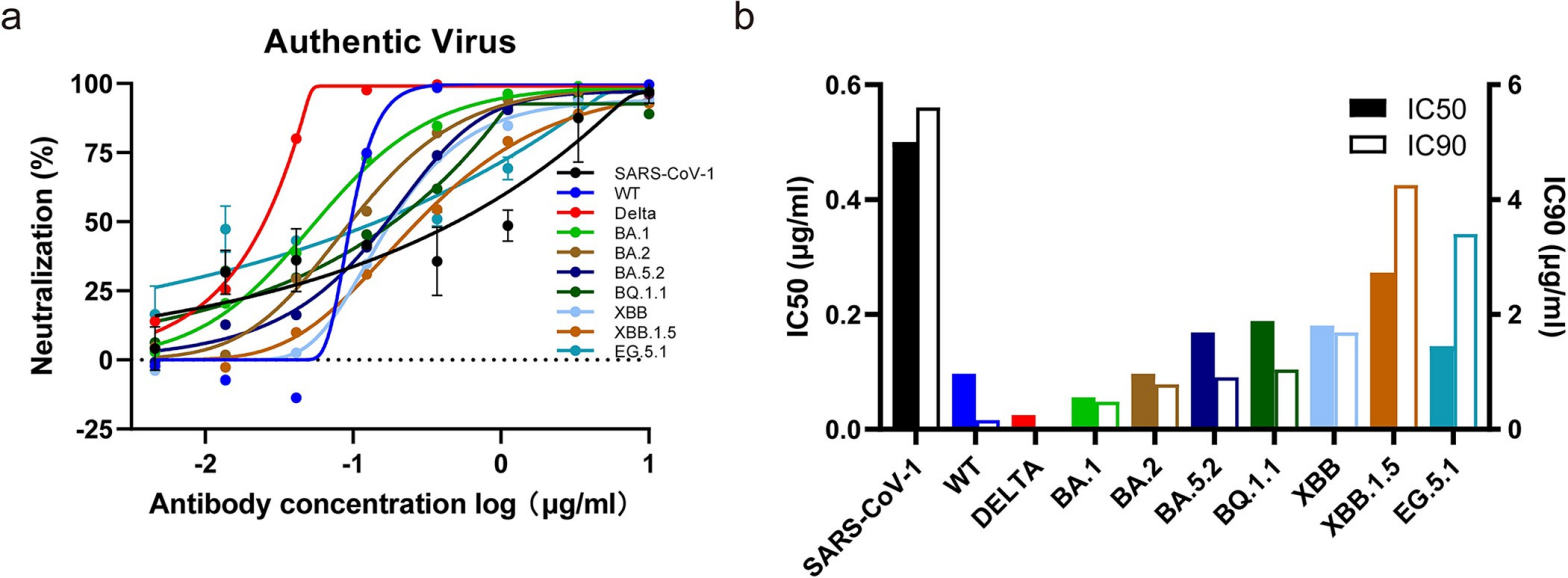
Broadly neutralizing activity of Tnb04-1 against authentic SARS-CoV-1 and SARS-CoV-2 variants. **a.** Actual neutralization curve of Tnb04-1 against diverse clinical isolates of SARS-CoV-1, SARS-CoV-2 WT, Delta, and various Omicron subvariants BA.1, BA.2, BA.5.2, BQ.1.1, XBB, XBB.1.5, and EG.5.1, using focus reduction neutralization test (FRNT). **b.** The estimated IC50 and IC90 based on neutralization curves in the left panel. The results shown are representatives of two independent experiments and presented as mean ± SEM.

We next evaluated the capacity of Tnb04-1 in neutralizing diverse clinical isolates of SARS-CoV-2 variants and SARS-CoV-1 available to us, using focus reduction neutralization test (FRNT). Tnb04-1 exhibited broad neutralizing activity to all authentic viruses tested, with the strongest against Delta (IC50: 0.025 μg/ml and IC90: 0.047 μg/ml), followed by WT-D614G and Omicron subvariants such as BA.1, BA.2, BA.5.2, BQ.1.1, XBB, XBB.1.5, and EG.5.1 (Fig 4). The greatest reduction in neutralizing activity occurred to SARS-CoV-1 (IC50: 0.531 μg/ml and IC90: 5.699 μg/ml, which is about 21- and 121-fold weaker compared to the Delta variant (Fig 4). Notably, the estimated neutralizing activity of Tnb04-1 was generally weaker to the authentic viruses compared to the corresponding pseudoviruses (S2 Fig). This discrepancy might be attributed to differences in assay systems; multiple rounds of replications in live virus vs. single-cycle entry in pseudovirus and Vero-E6 TMPRSS2 cells in live virus assay vs. HEK293T-hACE2 cells in pseudovirus assay. Regardless, these results demonstrate that Tnb04-1 is a broadly neutralizing nanobody, effective against a wide range of authentic clinical viral isolates.

### Structure basis and mechanism of action for the broad and potent neutralization by Tnb04-1

**Fig 5:**
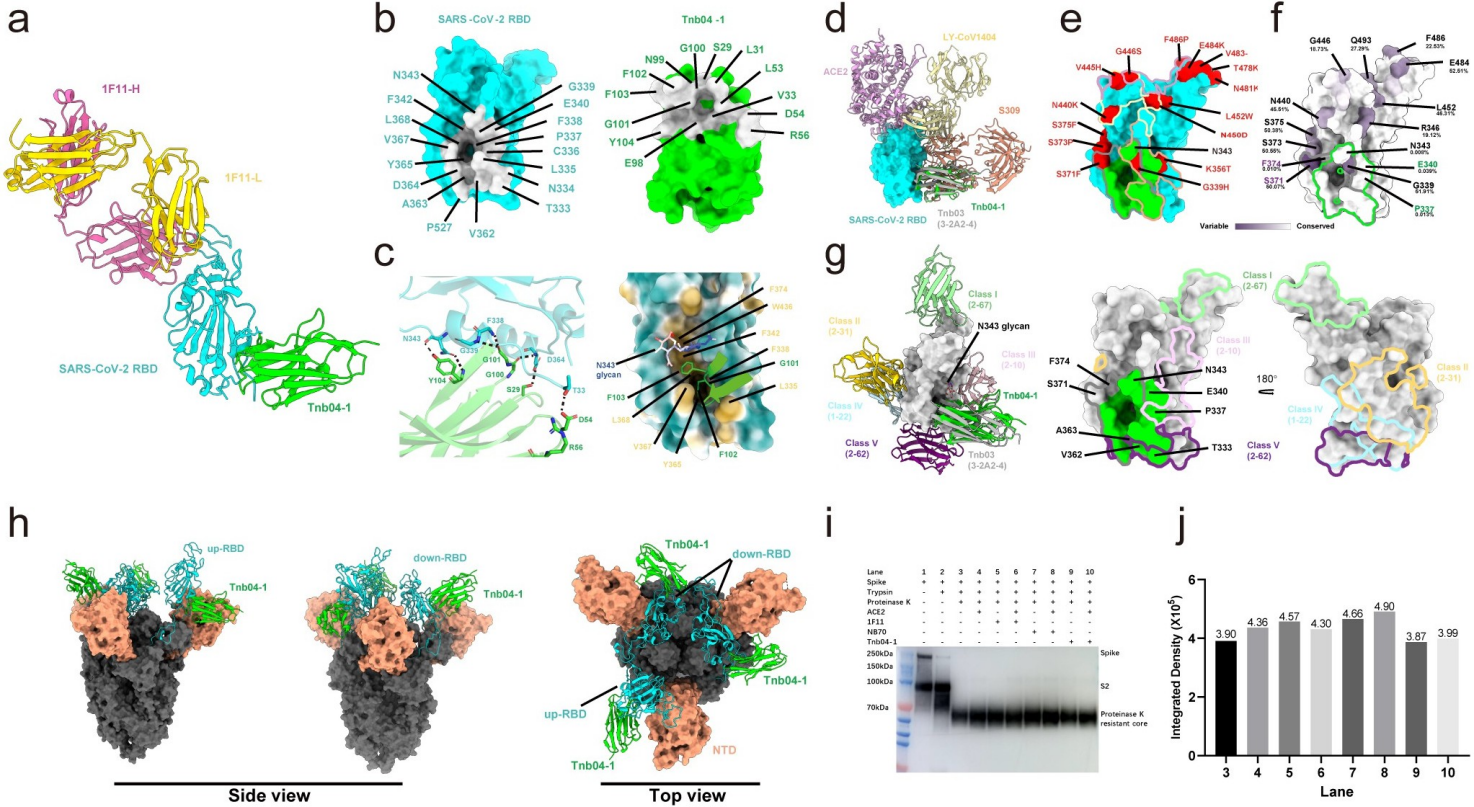
Structure basis and mechanism of action for the broadly neutralization by Tnb04-1. **a**. Overall crystal structure of the Tnb04-1 (green), SARS-CoV-2 RBD (cyan), and 1F11 fab (yellow and pink) complex. **b**. The epitope and paratope of Tnb04-1, highlighted in light gray in the context of RBD (cyan) and Tnb04-1 (green) and the involved residues are indicated. **c**. Molecular interaction between Tnb04-1 and SARS-CoV-2 RBD. The hydrogen bond interaction is shown on the left and the hydrophobic interaction on the right. The residues involved are indicated. **d.** The binding model of Tnb04-1 (green), Tnb03 (3-2A2-4) (gray), S309 (dark salmon), LY-CoV1404 (yellow), and ACE2 (purple) to SARS-CoV-2 RBD (cyan). **e.** The footprints of Tnb04-1 (green) on RBD, relative to that of ACE2 (purple) and control antibodies. Residue mutations found in BA.2.86 variant are highlighted in red. **f** Superimpose of variable and conserved residues on RBD structure derived from over 16 million sequences in the GISAID database collected from December 2019 to January 2024. Conserved residues are in white and variable residues in purple, and extent of which are in indicated by color gradient. Tnb04-1 gained two highly conserved epitope residues E340 and P337 (green) while excluded two relatively variable residues S371 and F374 (purple), compared to control Tnb03 (3-2A2-4). **g.** The epitope comparison between Tnb04-1, Tnb03 (3-2A2-4) and the five classes antibodies reported by Xiang et al [47] **h.** The simulated model of three Tnb04-1 bind to partially open SARS-CoV-2 S trimer (one RBD is open). Tnb04-1 is in green, RBD in cyan, NTD in dark salmon, and rest of the spike in dark gray. **i.** Detection of proteinase K-resistant core on a western blot probed with a rabbit anti-S2 polyclonal antibody. Various experimented conditions are indicated above. **j.** The integrated density of proteinase K-resistant core under each experimental condition detected and calculated by ImageJ.

To investigate the structural basis of Tnb04-1-mediated broad and potent neutralization, we determined the crystal structure of Tnb04-1 complexed with SARS-CoV-2 wildtype RBD at 2.3 Å resolution. This structure was determined in conjunction with the Fab fragment of P2C-1F11, an antibody isolated from a SARS-CoV-2 convalescent patient and known for its propensity to form high-quality crystals [46]. Our structural analysis revealed that Tnb04-1 binds to its epitope at the bottom of RBD, located between the cryptic and outer face, and distinctively away from the binding site of P2C-1F11 Fab (Fig 5A). Tnb04-1 epitope covers 683.6 Å^2^ surface area, involving 17 residues largely located in the N-terminal core structure of RBD (Fig 5B). The Tnb04-1 paratope consists of 13 residues, with a major contribution from seven residues in CDR3, complemented by three residues each from CDR1 and CDR2 (Fig 5B). At the binding interface, Tnb04-1 forms seven hydrogen bonds with the RBD, mediated by Tnb04-1 residues S29, D54, G100, G101, and Y104, and RBD residues T333, D364, F338, G339, and N343 (Fig 5C). CDR3 residue G101 forms dual interactions with both F338 and G339, whereas Y104 interacted with N343. Particularly, CDR3 residues F102 and F103 penetrate into a hydrophobic pocket, comprised of residues L335, F338, F342, Y365, V367, and L368, and form multiple hydrophobic interactions through their benzyl groups on the side chain (Fig 5C).

We then superimposed ACE2, control antibodies LY-CoV1404, S309, and Tnb03 (3-2A2-4) [28, 42, 43] onto the SARS-CoV-2 RBD-Tnb04-1 complex (Fig 5D). This reveals that Tnb04-1 binds outside the ACE2-binding region without causing steric hindrance, indicating a neutralization mechanism independent of direct ACE2 competition. Tnb04-1 overlaps the most with Tnb03 (3-2A2-4), followed by S309, and not at all with LY-CoV1404 (Fig 5D and 5E). Of its 17 epitope residues, 15 overlap with Tnb03 (3-2A2-4) (T333, N334, L335, C336, F338, G339, F342, N343, V362, A363, D364, Y365, V367, L368, and P527) whereas 8 with S309 (T333, N334, L335, C336, P337, G339, E340, and N343). This is not surprising given the sequence homology between Tnb04-1 and Tnb03 (3-2A2-4), differing in only five (Q1E, E16G, Q50R, S56R, and T116S) of the total 116 residues (S1 Fig). Compared to Tnb03 (3-2A2-4), Tnb04-1 approaches its epitope with a 21.2° shift towards the outer face of RBD (S5A and S5B Fig), which led to gain two conserved epitope residues (P337 and E340) on the outer face of RBD while excluding two relatively variable residues (S371 and F374) on the inner face (Fig 5F). Compared to Tnb03 (3-2A2-4) paratope, Tnb04-1 incorporates an additional residue R56 while losing the residue Q1 (Figs S1 and 5B). R56, despite its proximity to epitope residue T333 (3.9 Å), does not form a direct hydrogen bond. Regardless, Tnb04-1 epitope residues are highly conserved, reaching 96.60% among SARS-CoV-2 and 88.03% among sarbecoviruses among over 16 million RBD sequences submitted to GISAID. The only exception is G339 with 50.90% conservation (Fig 5F) although no obvious impact was found on Tnb04-1, suggesting the contribution of G339 to Tnb04-1 binding is rather small. We then performed an epitope comparison between Tnb04-1, Tnb03 (3-2A2-4), and the major classes of broadly neutralizing epitopes on the RBD, as previously reported by Xiang et al [47]. As shown in Fig 5G, Tnb04-1 shares one overlapping residue (P337) with the class III epitope and three overlapping residues (T333, V362, and A363) with class V. Similarly, Tnb03 (3-2A2-4) shares the same three residues (T333, V362, and A363) with class V. Together, the conserved epitope and unique angle of approach to its epitope provide structure basis for broadly neutralizing activity of Tnb04-1 against diverse sarbecoviruses tested.

To further investigate mechanism of action of Tnb04-1, we simulated its binding to a partially open SARS-CoV-2 spike (S) trimer, comprising one ’up’ and two ’down’ RBDs (PDB: 8a99). Tnb04-1 was able to engage all RBD conformations, suggesting an indirect modulation of the interaction between RBD and the ACE2 receptor (Fig 5G). We then studied the impact of Tnb04-1 on the formation of a proteinase K-resistant core, a surrogate for transitioning of spike trimer from a pre-hairpin intermediate to a six-helix bundle required for viral-cell fusion [28]. Specifically, equal amounts of spike trimer were incubated with Tnb04-1, control antibody P2C-1F11, or NB70 in the presence or absence of ACE2, followed by trypsin and proteinase K digestion. The resultant proteinase K-resistant core was detected as a 70 kDa band on a western blot probed with a rabbit anti-S2 polyclonal antibody. The presence of ACE2 substantially enhanced the formation of proteinase K-resistant core (Lane 4 vs. Lane 3 in Fig 5I and 5j), consistent with the role of ACE2 in facilitating spike conformational change. Notably, Tnb04-1 substantially reduced this core formation to the baseline levels, irrespective of ACE2 presence (Lane 3 vs. Lane 9 and Lane 10, Fig 5I and 5J). This result indicates that Tnb04-1, although not directly competing with ACE2 for binding to RBD, might indirectly affect their interaction. Given the sequence and structural similarities between Tnb04-1 and Tnb03 (3-2A2-4), it is plausible that Tnb04-1 shares a similar action mechanism, where it interferes with structural changes in the RBD necessary for ACE2 binding [28]. Intriguingly, control antibodies P2C-1F11 and NB70 substantially increase the core formation in the absence of ACE2 (Lanes 5 vs.3 and 7 vs. 3, Fig 5J), suggesting that their binding to the RBD may prematurely induce an allosteric opening of the spike trimer, facilitating six-helix bundle formation even prior to target cell contact [28].

### Combined effect of 5 paratope mutations for the enhanced activity of Tnb04-1

**Fig 6:**
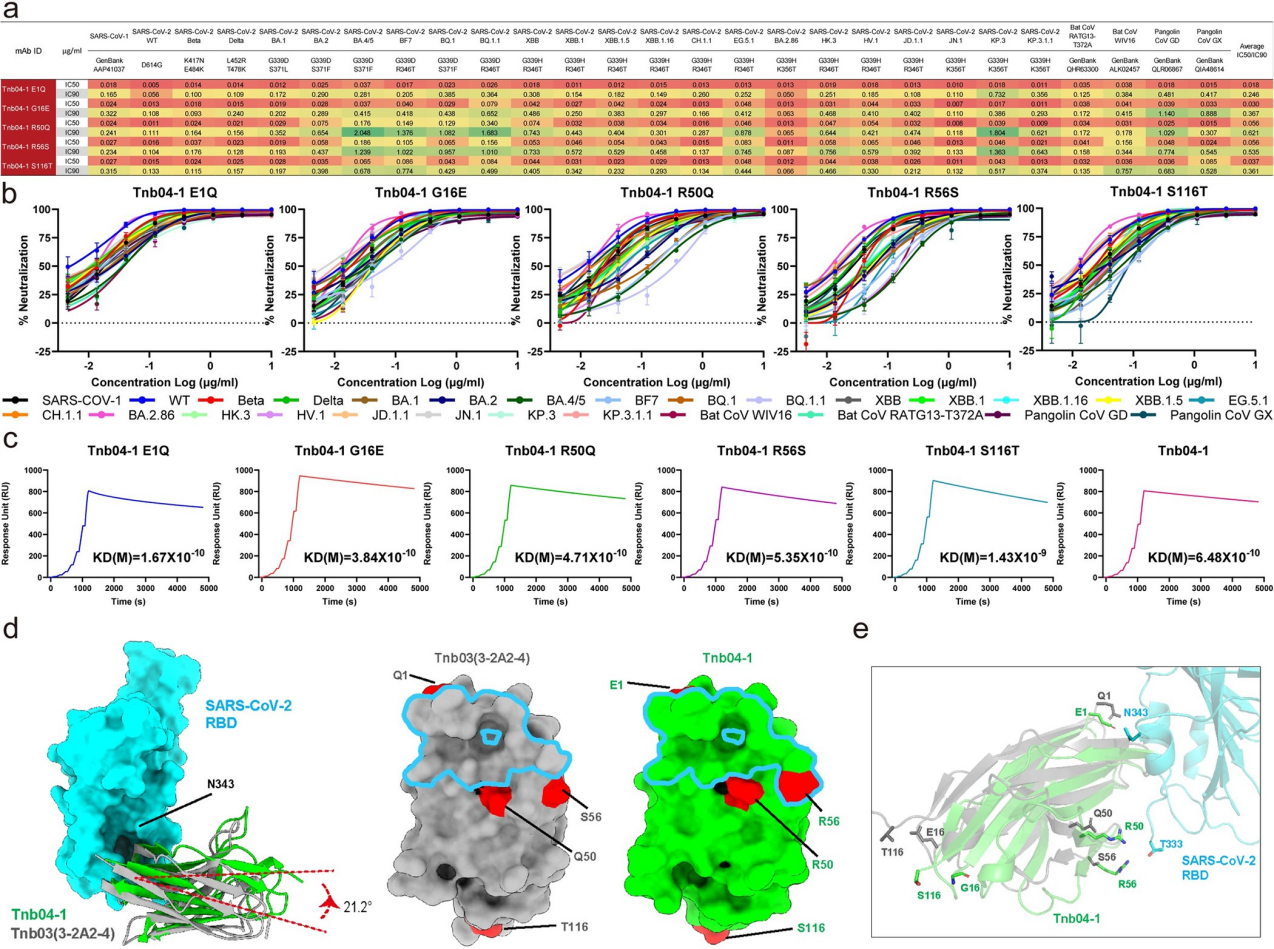
Combined effect of five paratope mutations for the enhanced activity of Tnb04-1. **a.** Neutralizing activity (IC50 and IC90) of the five Tnb04-1 mutants against a panel of 27 diverse pseudoviruses. **b.** Neutralization curves of the five Tnb04-1 mutants, from which IC50 and IC90 are estimated. **c.** Binding kinetics of the five Tnb04-1 mutants compared to the wildtype Tnb04-1. **d.** Tnb04-1 (green) shows a 21.2° shift towards the outer face of the RBD relative to Tnb03 (3-2A2-4) (gray). Paratope and epitope residues for Tnb03 (left) and Tnb04-1 (right) are highlighted in red. **e.** Comparison of molecular interactions between Tnb04-1 and Tnb03 (3-2A2-4) with the SARS-CoV-2 RBD, highlighting paratope substitutions in green for Tnb04-1 and in gray for Tnb03 (3-2A2-4).

To further investigate the contribution of each of the 5 paratope substitutions to the enhanced activity of Tnb04-1, we first generated five mutant forms of Tnb04-1, each containing a single substitution reverting it to the corresponding residue in Tnb03 (3-2A2-4) at the aforementioned five locations. We then evaluated the impact of these changes on neutralizing activity against the same panel of pseudoviruses and binding affinity to the wild-type SARS-CoV-2 RBD using SPR. As shown in Fig 6A and 6B, none of these single substitutions resulted in substantial changes in neutralization breadth or potency, suggesting that the combined effect of all five mutations plays a crucial role. Similar to the original Tnb04-1, Tnb04-1 E1Q exhibited an IC50 of 0.018 μg/ml and an IC90 of 0.246 μg/ml against the panel of pseudoviruses, while Tnb04-1 E16G has an IC50 of 0.03 μg/ml and an IC90: 0.367 μg/ml. Tnb04-1 S116T displayed an IC50 of 0.037 μg/ml and an IC90: 0.361 μg/ml. In addition, Tnb04-1 R50Q and Tnb04-1 R56S demonstrated only minor reductions in overall average IC50 and IC90, with more pronounced decline against earlier Omicron variants such as BA.4/5, BF7, BQ.1, BQ.1.1. This is likely due to Q50R’s proximity to or the location of S56R within the CDR2 region of the antibody.

Similarly, no significant differences in binding affinity were observed between the five mutant forms and wild-type Tnb04-1, suggesting that affinity alone does not account for the improved breadth and potency of Tnb04-1 over Tnb03 (3-2A2-4) (Fig 6C). Structural analysis suggests that the gain of two conserved epitope residues (P337 and E340) and the loss of two variable residues (S371 and F374) compared to Tnb03 likely contributed to the enhanced breadth and potency of Tnb04-1, which resulted in a 21.2° shift towards the outer face of the RBD (Figs 6D, S5A and S5). This shift may also be reinforced by Q50R and S56R substitutions near or within the paratope of Tnb04-1 (Fig 6D), creating additional, albeit weak, interactions with the epitope residue T333 on the RBD (Fig 6E).

### Tnb04-1 confers protection against SARS-CoV-2 Omicron XBB.1.5 in Syrian hamsters

**Fig 7:**
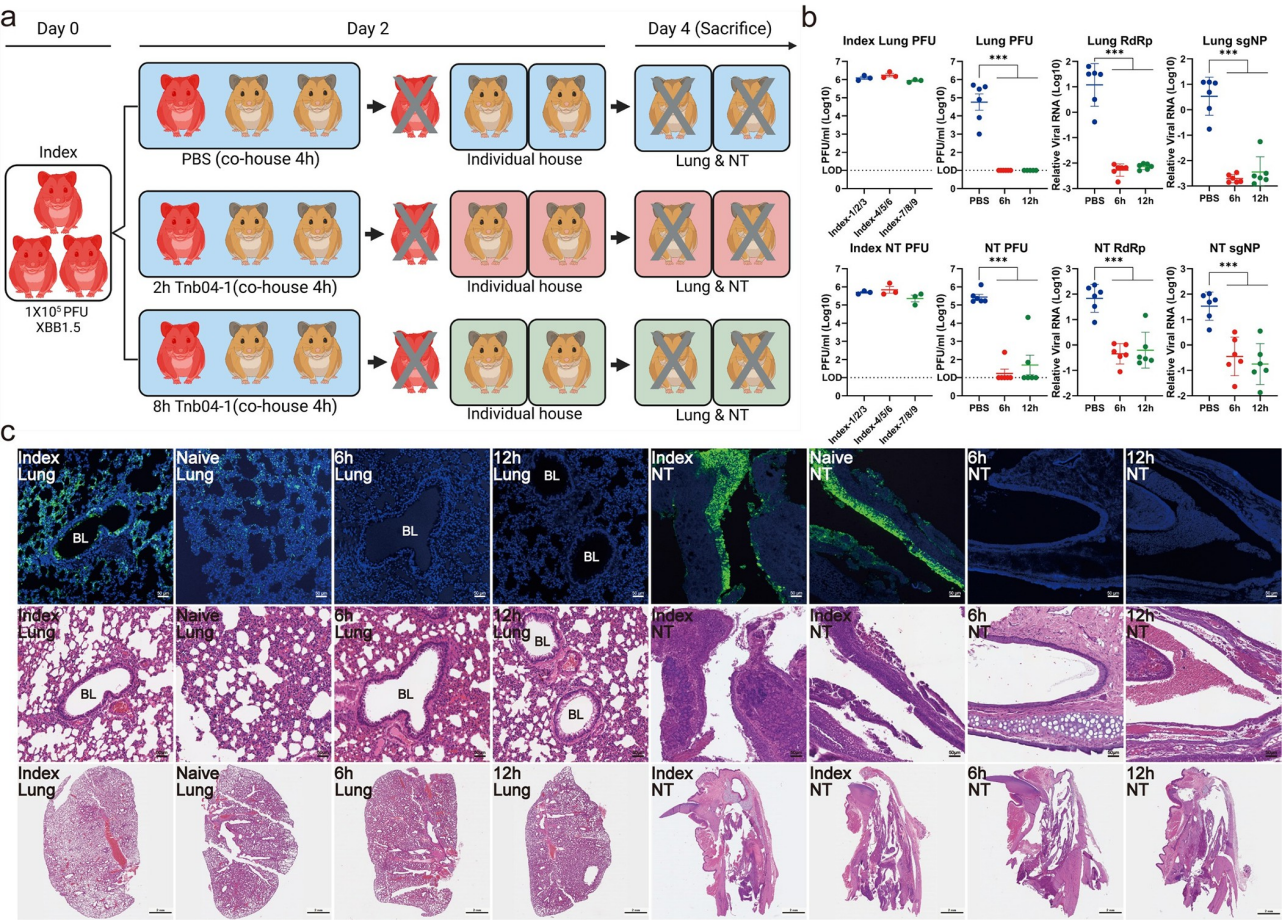
Tnb04-1 prophylaxis protecting Syrian hamsters from contact transmission of Omicron variant XBB.1.5. **a.** Experimental schedule for nanobody prophylaxis. On day 0, a total of 9 index hamsters were infected with Omicron variant XBB.1.5. On day 2, naïve hamsters were intranasally inoculated with 4.5 mg/kg Tnb04-1 or PBS for 2 or 8 hours before co-housing with infected index hamsters for 4 hours. After which, the index hamsters were immediately sacrificed to collect lung tissues and nasal turbinate (NT) whereas each naïve hamster was separated and continuously housed in a new single cage for additional 2 days. On day 4, all naïve hamsters were sacrificed for collecting lung tissues and NT. The image was created by BioRender.com. **b.** Viral burden in lung tissue and NT measured by plaque-forming units (PFUs) and a quantitative RT-PCR specific to genomic RdRp and sub-genomic NP. The RNA copies were measured in triplicates, and the data was presented as the means ± SEM. Statistics were generated using one-way ANOVA followed by Tukey’s multiple comparisons test (***P < 0.001). **c.** Representative images of infected cells (green) in lung tissues and NT from each group, as determined by anti-NP immunofluorescence staining. Cell nuclei were counterstained with DAPI (blue). Representative histopathology sections of the lung tissues from each group stained with hematoxylin and eosin (H&E). BL bronchiolar lumen. Scale bars: 50 μm and 2 mm as indicated.

We investigated the protective potential of Tnb04-1 in Syrian hamster model against contact transmission [48, 49]. On day 0, we intranasally infected nine index hamsters with the authentic XBB.1.5 variant (Fig 7A). On day 2 post-infection, groups of three index hamsters were co-housed with six naive hamsters pre-treated intranasally with either control PBS for 2 hours or Tnb04-1 for 2 or 8 hours. The index hamsters were euthanized right after co-housing, while the naive hamsters continued to be monitored until day 4 when they were sacrificed. Lung and nasal turbinate (NT) tissues were harvested for viral load and histopathological analysis.

In the index animals, the average infectious viral titers in the lung and NT were 1.3x10^6^/ml and 5.3x10^5^/ml plaque-forming units (PFUs), respectively, confirming effective infection with XBB.1.5 (Fig 7B). Control PBS-treated animals showed average PFUs of 2.1x10^5^/ml in lungs and 3.9x10^5^/ml in NT, indicating successful virus transmission and replication from the index hamsters. Interestingly, PFU variance in the lungs was more pronounced than in the NT, perhaps reflecting varying stages of infection in the upper and lower respiratory tracts. In stark contrast, Tnb04-1-treated animals exhibited no detectable PFUs in the lungs, regardless from the 6 hours (2h treated + 4h co-house) (red) or 12 hours (8h treated + 4h co-house) (green) scarified groups post administration. In the corresponding NT, only one in the 6h and two in the 12h group had detectable PFU (Fig 7B). Moreover, Tnb04-1-treated animals showed approximately a 3-log reduction in RdRp and sgNP RNAs in the lungs and about 2-log reduction in the NT compared to PBS controls (Fig 7B). Notably, RdRp and sgNP levels in the NT were about tenfold higher than in the lung, aligning with observations of higher Omicron replication efficiency in the upper respiratory tract [50]. Immunofluorescence staining of lung and NT tissues showed numerous viral particles in both index and PBS-treated groups but sporadically detected in Tnb04-1-treated animals (Figs 7C and S6). Additionally, lung and NT tissues from index and PBS groups displayed moderate damage, together with marked infiltration and inflammatory cells (Fig 7C). Collectively, these findings demonstrate effective protection of Tnb04-1 against contact transmission in the Syrian hamster model.

## Discussion

We have isolated five exceptionally broad and potent nanobodies from an immunized alpaca, effective against a range of sarbecoviruses. Tnb04-1, the best among these, showed remarkable neutralizing activity not only to the newly emerged and highly divergent Omicron subvariants EG.5, HK3, HV.1, JD.1.1, BA.2.86, JN.1, KP.3, and KP.3.1.1 but also to SARS-CoV-1, and coronaviruses from bats and pangolins that utilize the receptor ACE2. This impressive in vitro activity translated into robust protection against both contact and respiratory transmission of the Omicron XBB.1.5 subvariant in a Syrian hamster model of SARS-CoV-2 infection [48, 51]. Crystal structure analysis of Tnb04-1 revealed a highly conserved epitope consisting of a hydrophobic pocket between the cryptic and outer face of the RBD, distinct from the ACE2 binding site. The conserved epitope residues and the unique binding pose provide structural basis for Tnb04-1 in overcoming the large number of mutations in new Omicron subvariants. Mechanistically, Tnb04-1 engages with various configurations of RBDs and substantially reduces the formation of a proteinase K-resistant core. In conclusion, Tnb04-1 emerges as an exceptionally broad and potent nanobody, presenting a promising candidate for disrupting the cycle of immune selection and viral escape through effectively blocking respiratory acquisition and transmission of diverse sarbecoviruses.

The exceptional breadth and potency of Tnb04-1 are attributed to its conserved epitope and binding pose, which are rare among published antibodies and nanobodies. Through extensive search and structural comparison across various databases, we identified only one nanobody (Tnb03/3-2A2-4) and three antibodies (47D11, Beta-49, and Beta-50) with similar binding properties to Tnb04-1. Despite substantial sequence and structure similarity with nanobody Tnb03 (3-2A2-4), Tnb04-1 approaches its epitope with 21.2° shift towards the outer face of RBD, enabling Tnb04-1 to target more conserved epitope and thus with broader and stronger activity over Tnb03 (3-2A2-4). Furthermore, the three antibodies (47D11, Beta-49, and Beta-50) also recognize the same hydrophobic pocket through CDRH3 residues although 47D11 mediates by paratope residues W102 and F103 while Beta-49 and Beta-50 by W102 (S5C Fig). More importantly, like Tnb04-1, these three antibodies also demonstrated cross-neutralization to SARS-CoV-1, multiple variants of SARS-CoV-2, and bat and pangolin coronaviruses, although the panel of viruses tested were substantially smaller than the current study [52, 53]. Nevertheless, our results highlight the epitope of Tnb04-1 and the surrounding residues are highly conserved. Detailed characterization of these antibodies will provide deeper insights into their ontogeny and potential ways of inducing similar and more effective protection against the circulating and future variants.

While our study presents valuable insights, it also has some inherent limitations. First, as the immune response in alpaca is likely different from that in human, the broadly neutralizing nanobodies and their epitopes may not perfectly align with human responses. However, the discovery of antibodies like Beta-49 and Beta-50 in convalescent humans and 47D11 in transgenic mice testify the relevance of Tnb04-1-like antibodies and epitopes they recognize. Thus, the conserved hydrophobic pocket in the RBD is an effective and accessible target not just in alpacas, but also in humans and transgenic mice. Second, Tnb04-1 protection experiments were limited to 6h and 12h after intranasal administration. Extending the incubation time before viral challenge would be necessary to better understand the duration of protection. Finally, our in vivo experiments were performed exclusively in the Syrian hamster model of SARS-CoV-2 infection and transmission, which may not fully mimic the human protection to respiratory SARS-CoV-2 infection. Future studies involving non-human primates and/or human subjects would be required to verify and validate the protection results.

## Materials and Methods

### Ethics statement

The animal study was approved by the Committee on the Use of Live Animals in Teaching and Research (CULATR 5518–20) of the University of Hong Kong (HKU).

### Cell lines

HEK293T cells (ATCC, CRL-3216) were maintained at 37°C in 5% CO_2_ in Dul-becco’s minimal essential medium (DMEM) containing 10% (v/v) heat-inactivated fetal bovine serum (FBS) and 100 U/ml penicillin–streptomycin. HEK293T cells expressing hACE2 were kindly provided by Dr. Zheng Zhang from Shenzhen Third People’s Hospital, Shenzhen, China and maintained at complete DMEM medium supplemented with 1 μg/ml puromycin (Sigma). We transfected lentivirus vector pLVX-cRL, packaging vector psPAX2 and envelope vector pMD2G (NIH) into HEK293T cells with PEI (Sigma, St. Louis, MO, USA) to produce cRL lentivirus particle. Huh-7 cells were infected with cRL lentivirus for 48h before the medium was replaced with 5 μg/ml puromycin containing fresh medium for selecting the positive colonies for 7 days before use. FreeStyle 293F cells (Thermo Fisher Scientific, R79007) were maintained at 37°C in 5% CO_2_ in SMM 293-TII expression medium (Sino Biological, M293TII). Sf9 cells (ATCC) were maintained at 27°C in Sf-900 II SFM medium. Hi5 cells (ATCC) were maintained at 27°C in SIM HF medium.

### Expression and purification of recombinant proteins

The genes encoding the receptor-binding domain (RBD) of SARS-CoV-2 prototype strain (GenBank: MN908947.3), SARS-CoV-2 variant BF7 (GISAID: EPI_ISL_15429967), SARS-CoV-2 variant XBB.1.5 (GISAID: EPI_ISL_16283160) were constructed as previously described [16, 28]. All constructs contained 6×His tag sequence at C-terminal for purification purpose. The recombinant RBD were expressed by transfecting each of the above expressing plasmid into the FreeStyle 293F cells and purified from the cell culture supernatant by Ni-NTA resin (QIAGEN 30230) followed by gel filtration chromatography. For crystal structure analysis, the recombinant RBD of the prototype strain was expressed using the Bac-to-Bac Baculovirus System (Invitrogen). Specifically, the gene fragment encoding the RBD (residues Arg319 to Lys529) and containing the gp67 secretion signal sequence and a C-terminal 6×His tag sequence was inserted into pFastBac-Dual vectors (Invitrogen) and transformed into DH10 Bac component cells. The recombinant bacmid was extracted and further transfected into Sf9 cells using Cellfectin II Reagents (Invitrogen). The recombinant viruses were harvested from the transfected supernatant, amplified to generate high-titer virus stock, and then used to infect Hi5 cells for protein expression. Secreted RBD was harvested from the supernatant, captured by Ni-NTA Sepharose (GE Healthcare), and purified by gel filtration chromatography.

### Immunization of alpaca, construction of yeast display VHH library, and isolation of VHH yeasts specific for spike trimers of SARS-CoV-1 and SARS-CoV-2 Omicron variant BA.4/5

The alpaca experiment protocol involving immunization, collection of blood samples, and construction of the VHH library was performed as previously reported [28, 54]. To select VHH yeasts specific for the spike (S) trimers of SARS-CoV-1 and SARS-CoV-2 Omicron variant BA.4/5, the yeast library was firstly cultivated in SDCAA media at 30°C for 48 h to reach the exponential growth phase and then transferred to SGCAA media for induction of VHH expression at 20°C for 36 h. Yeast clones were enriched through a single round of MACS biopanning, and followed by an additional round of FACS biopanning against S trimers of SARS-CoV-1 and SARS-CoV-2 Omicron BA.4/5. Specifically, the induced yeast library was incubated with 100nM S timer of SARS-CoV-1 on ice for 30 min. The yeast library was washed thrice with cold PBS containing 1%FBS and then incubated with streptavidin microbeads on ice for 10 min. The mixture was passed through the LS column and the S trimer-positive yeasts were harvested for further culture and induction of VHH expression. Subsequently, the induced yeasts were further incubated with 100nM S-6P timer of SARS-CoV-2 Omicron BA.4/5 on ice for 30 min. After extensive wash with cold PBS+1%FBS, the yeast clones were incubated with HA-Tag (6E2) mouse monoclonal antibody conjugated with Alexa Fluor 488 (Cell Signaling 2350S, 1:100 dilution) and streptavidin conjugated with PE Conjugate (eBioscience 12-4317-87, 1:200 dilution) on ice for 30min. The yeast clones were washed three times with cold PBS containing 1% FBS before being analyzed by FACS using Aria II (BD Biosciences). Positive yeast clones for both Alexa Fluor 488 and PE Conjugate were sorted and utilized for nanobody cloning.

### Molecular cloning and expression of nanobody and IgG

The gene fragment encoding various VHHs were amplified from the sorted yeast clones and integrated into VHH expression vector in conjunction with human IgG1 Fc fragment. The published SARS-CoV-2 mAbs were synthesized according to the sequences released in Protein Data Bank (PDB). To produce antibodies, equal amounts of paired heavy- and light-chain plasmids or heavy chain alone (nanobody-Fc) were transfected into the HEK293F cells (Life Technologies, Carlsbad, CA, USA) by PEI. Five days later, the culture supernatant containing antibodies was collected and captured with protein A microbeads (GenScript, Piscataway, NJ, USA) according to the manufacturer’s protocol. Antibody-beads complexes were concentrated using the magnetic separation rack and the captured antibodies were eluted from beads with an elution buffer (0.3 M glycine, pH 2.0) into a neutralization buffer (1 M Tris-HCl, pH 8.0), followed by dialyzing into phosphate-buffered saline (PBS). The exact antibody concentration was estimated by nanodrop 2000 Spectrophotometer (Thermo Scientific).

### Cell surface staining

HEK 293T cells were transfected with expression plasmids encoding the spikes of SARS-CoV-1 or SARS-CoV-2 Omicron BA.4/5, and incubated at 37°C for 24 h. Cells were then digested from the plate with trypsin and distributed onto 96-well plates. Cells were washed twice with 200μL staining buffer (PBS with 1% heated-inactivated fetal bovine serum) between each of the following steps. First, cells were stained with each nanobody-Fc (20 μg/mL) at 4°C for 20 min, followed by anti-human IgG Fc-PE (Biolegend 410708, 1:200 dilution) at 4°C for another 20 min. After extensive washing, the cells were resuspended and analyzed using BD LSRFortassa (BD Biosciences, USA) and FlowJo 10 software (FlowJo, USA). The published monoclonal antibodies LY-COV1404, S309, and CR3022, along with human antibody P2C-1F11 and nanobody NB70-Fc previously isolated by our group[28], were used as controls. HEK 293T cells with mock transfection were stained as a background control. The number of positive cells in the selected gates was calculated.

### Phylogenetic tree and genetic analysis of nanobodies

Isolated nanobodies were analyzed and compared with those previously isolated by our group [28] and those from the CoV-AbDab database (https://opig.stats.ox.ac.uk/webapps/covabdab/) updated on 21st March, 2023. Multiple sequence alignment was conducted by MAFFT (version 7.490) after removing duplicates. Maximum likelihood phylogenetic tree was generated using IQ-TREE (version 2.0.3), which contains ModelFinder to select the most accurate model as well as UFBoot to calculate unbiased branch support values with 1000 bootstrap replicates. The tree file was then visualized by an online tool iTOL (version 6.8.1) (https://itol.embl.de/). The IMGT/V-QUEST program (http://www.imgt.org/IMGT_vquest/vquest) was used to analyze the germline gene and the loop lengths of complementarity determining region 3 (CDR3) of each nanobody.

### Pseudoviruses production and neutralizing assay

Production of pseudoviruses and neutralizing assay were performed as previously reported [16]. The various spike sequences used in the analysis are previously reported from the prototype up to Omicron subvariants BA.4/5 [28]. Additional spikes used in the current study including the following. The Omicron BF7 variant (Pango lineage BF7, GISAID: EPI_ISL_15429967) was constructed with 32 mutations in the spike including T19I, 24-26del, A27S, 69-70del, G142D, V213G, G339D, R346T, S371F, S373P, S375F, T376A, D405N, R408S, K417N, N440K, L452R, S477N, T478K, E484A, F486V, Q498R, N501Y, Y505H, D614G, H655Y, N679K, P681H, N764K, D796Y, Q954H, and N969K. The Omicron BQ.1 variant (Pango lineage BQ.1, GISAID: EPI_ISL_15458271) was constructed with 33 mutations in the spike including T19I, 24-26del, A27S, 69-70del, G142D, V213G, G339D, S371F, S373P, S375F, T376A, D405N, R408S, K417N, N440K, K444T, L452R, N460K, S477N, T478K, E484A, F486V, Q498R, N501Y, Y505H, D614G, H655Y, N679K, P681H, N764K, D796Y, Q954H, and N969K. The Omicron BQ.1.1 variant (Pango lineage BQ.1.1, GISAID: EPI_ISL_15458263) was constructed with 34 mutations in the spike including T19I, 24-26del, A27S, 69-70del, G142D, V213G, G339D, R346T, S371F, S373P, S375F, T376A, D405N, R408S, K417N, N440K, K444T, L452R, N460K, S477N, T478K, E484A, F486V, Q498R, N501Y, Y505H, D614G, H655Y, N679K, P681H, N764K, D796Y, Q954H, and N969K. The Omicron XBB variant (Pango lineage XBB, GISAID: EPI_ISL_15601178) was constructed with 39 mutations in the spike including T19I, 24-26del, A27S, V83A, G142D, 145del, H146Q, Q183E, V213E, G339H, R346T, L368I, S371F, S373P, S375F, T376A, D405N, R408S, K417N, N440K, V445P, G446S, N460K, S477N, T478K, E484A, F486S, F490S, Q498R, N501Y, Y505H, D614G, H655Y, N679K, P681H, N764K, D796Y, Q954H, and N969K. The Omicron XBB.1 variant (Pango lineage XBB.1, GISAID: EPI_ISL_15596825) was constructed with 40 mutations in the spike including T19I, 24-26del, A27S, V83A, G142D, 145del, H146Q, Q183E, V213E, G252V, G339H, R346T, L368I, S371F, S373P, S375F, T376A, D405N, R408S, K417N, N440K, V445P, G446S, N460K, S477N, T478K, E484A, F486S, F490S, Q498R, N501Y, Y505H, D614G, H655Y, N679K, P681H, N764K, D796Y, Q954H, and N969K. The Omicron XBB.1.5 variant (Pango lineage XBB.1.5, GISAID: EPI_ISL_16283160) was constructed with 40 mutations in the spike including T19I, 24-26del, A27S, V83A, G142D, 145del, H146Q, Q183E, V213E, G252V, G339H, R346T, L368I, S371F, S373P, S375F, T376A, D405N, R408S, K417N, N440K, V445P, G446S, N460K, S477N, T478K, E484A, F486P, F490S, Q498R, N501Y, Y505H, D614G, H655Y, N679K, P681H, N764K, D796Y, Q954H, and N969K. The Omicron XBB.1.16 variant (Pango lineage XBB.1.16, GISAID: EPI_ISL_17370520) was constructed with 41 mutations in the spike including T19I, 24-26del, A27S, V83A, G142D, 145del, H146Q,E180V, Q183E, V213E, G252V, G339H, R346T, L368I, S371F, S373P, S375F, T376A, D405N, R408S, K417N, N440K, V445P, G446S, N460K, S477N, T478R, E484A, F486P, F490S, Q498R, N501Y, Y505H, D614G, H655Y, N679K, P681H, N764K, D796Y, Q954H, and N969K. The Omicron CH.1.1 variant (Pango lineage CH.1.1, GISAID: EPI_ISL_18141861) was constructed with 45 mutations in the spike including T19I, L24del, P25del, P26del, A27S, G142D, K147E, W152R, F157L, N185D, I210V, V213G, G257S, G339H, R346T, S371F, S373P, S375F, T376A, D405N, R408S, K417N, N440K, K444T, G446S, L452R, L455F, F456L, N460K, S477N, T478K, E484A, F486S, Q498R, N501Y, Y505H, D614G, H655Y, N679K, P681H, N764K, D796Y, L858I, Q954H, N969K. The Omicron EG.5.1 variant (Pango lineage EG.5.1, GISAID: EPI_ISL_18219020) was constructed with 49 mutations in the spike including T19I, L24del, P25del, P26del, A27S, Q52H, R78K, V83A, G142D, Y144del, Y144del, Y145del, H146Q, Q183E, V213E, G252V, G339H, R346T, L368I, S371F, S373P, S375F, T376A, D405N, R408S, K417N, N440K, V445P, G446S, F456L, N460K, S477N, T478K, E484A, F486P, F490S, S494P, Q498R, N501Y, Y505H, D614G, H655Y, N679K, P681H, N764K, D796Y, Q954H, N969K, E1202Q. The Omicron BA.2.86 variant (Pango lineage BA.2.86, GISAID: EPI_ISL_18213025) was constructed with 60 mutations in the spike including ins16MPLF, T19I, R21T, L24del, P25del, P26del, A27S, S50L, H69del, V70del, V127F, G142D, Y144del, F157S, R158G, N211del, L212I, V213G, L216F, H245N, A264D, I332V, G339H, K356T, S371F, S373P, S375F, T376A, R403K, D405N, R408S, K417N, N440K, V445H, G446S, N450D, L452W, N460K, S477N, T478K, N481K, V483del, E484K, F486P, Q498R, N501Y, Y505H, E554K, A570V, D614G, P621S, H655Y, N679K, P681R, N764K, D796Y, S939F, Q954H, N969K, P1143L. The Omicron HK.3 variant (Pango lineage HK.3, GISAID: EPI_ISL_18381864) was constructed with 45 mutations in the spike including T19I, L24del, P25del, P26del, A27S, A28S, A29S, A30S, Q52H, V83A, G142D, Y144del, H146Q, Q183E, V213E, G252V, G339H, R346T, L368I, S371F, S373P, S375F, T376A, R408S, K417N, N440K, V445P, G446S, L455F, F456L, N460K, S477N, T478K, E484A, F486P, F490S, Q498R, N501Y, Y505H, H655Y, N679K, P681H, N764K, Q954H, N969K.The Omicron HV.1 variant (Pango lineage HV.3, GISAID: EPI_ISL_18510706) was constructed with 46 mutations in the spike including T19I, L24del, P25del, P26del, A27S, Q52H, V83A, G142D, Y144del, H146Q, F157L, Q183E, V213E, G252V, G339H, R346T, L368I, S371F, S373P, S375F, T376A, D405N, R408S, K417N, N440K, V445P, G446S, L452R, F456L, N460K, S477N, T478K, E484A, F486P, F490S, Q498R, N501Y, Y505H, D614G, H655Y, N679K, P681H, N764K, D796Y, Q954H, N969K. The Omicron JD.1.1 variant (Pango lineage HK.3, GISAID: EPI_ISL_18596500) was constructed with 60 mutations in the spike including ins16MPLF, T19I, R21T, L24del, P25del, P26del, A27S, S50L, H69del, V70del, V127F, G142D, Y144del, F157S, R158G, L212I, L216F, N211del, V213G, H245N, A264D, I332V, G339H, K356T, S371F, S373P, S375F, T376A, R403K, D405N, R408S, K417N, N440K, V445H, G446S, N450D, L452W, L455S, N460K, S477N, T478K, N481K, E484K, F486P, Q498R, N501Y, Y505H, E554K, A570V, D614G, P621S, H655Y, N679K, P681R, N764K, D796Y, S939F, Q954H, N969K, P1143L. The Omicron JN.1 variant (Pango lineage JN.1, GISAID: EPI_ISL_18543810) was constructed with 61 mutations in the spike including ins16MPLF, T19I, R21T, L24del, P25del, P26del, A27S, S50L, H69del, V70del, P1143L, V127F, G142D, Y144del, F157S, R158G, N211del, L212I, V213G, L216F, H245N, A264D, I332V, G339H, K356T, S371F, S373P, S375F, T376A, R403K, D405N, R408S, K417N, N440K, V445H, G446S, N450D, L452W, L455S, N460K, S477N, T478K, N481K, V483del, E484K, F486P, Q498R, N501Y, Y505H, E554K, A570V, D614G, P621S, H655Y, N679K, P681R, N764K, D796Y, S939F, Q954H, N969K. The Omicron KP.3 variant (Pango lineage KP.3, GISAID: EPI_ISL_19146026) was constructed with 63 mutations in the spike including T19I, R21T, L24S, P25del, P26del, A27S, S50L, H69del, V70del, V127F, G142D, Y144del, F157S, R158G, N211I, L212del, V213G, L216F, H245N, A264D, I332V, G339H, K356T, S371F, S373P, S375F, T376A, R403K, D405N, R408S, K417N, N440K, V445H, G446S, N450D, L452W, L455S, F456L, N460K, S477N, T478K, N481K, V483del, E484K, F486P, Q493E, Q498R, N501Y, Y505H, E554K, A570V, D614G, P621S, H655Y, N679K, P681R, N764K, D796Y, S939F, Q954H, N969K, V1104L, P1143L. The Omicron KP.3.1.1 variant (Pango lineage KP.3.1.1, GISAID: EPI_ISL_19272251) was constructed with 64 mutations in the spike including T19I, R21T, L24S, P25del, P26del, A27S, S31del, S50L, H69del, V70del, V127F, G142D, Y144del, F157S, R158G, N211I, L212del, V213G, L216F, H245N, A264D, I332V, G339H, K356T, S371F, S373P, S375F, T376A, R403K, D405N, R408S, K417N, N440K, V445H, G446S, N450D, L452W, L455S, F456L, N460K, S477N, T478K, N481K, V483del, E484K, F486P, Q493E, Q498R, N501Y, Y505H, E554K, A570V, D614G, P621S, H655Y, N679K, P681R, N764K, D796Y, S939F, Q954H, N969K, V1104L, P1143L.

For sarbecoviruses that use ACE2 as an entry receptor, the pseudovirus was constructed with cDNAs encoding the S trimer of the following strains: SARS-CoV-1 spike (GenBank AAP41037), Pangolin CoV GD spike (GenBank QLR06867.1), Pangolin CoV GX spike (GenBank QIA48614.1), Bat SARS-like coronavirus WIV16 spike (GenBank ALK02457.1), and Bat SARS-like RaTG13 spike (GenBank QHR63300.2). The full-length genes encoding these spikes variants were synthesized by Genwiz Inc., China and verified by sequencing.

Pseudoviruses were generated by co-transfecting HEK 293T cells with human immunodeficiency virus backbones expressing firefly luciferase (pNL4-3-R-E-luciferase) and pcDNA3.1 vector encoding either prototype or variant S proteins. Viral supernatant was collected 48 h or 72 h later, centrifuged to remove cell lysis, and stored at −80°C until use. Viral infectious titers were measured by luciferase activity in the HEK293T-hACE2 cells using Bright-Lite Luciferase Assay System (Vazyme). GloMax Discover Microplate Reader (Promega) was used for measuring luciferase activity. Neutralization assays were performed by incubating pseudoviruses with serial dilutions of purified nanobodies or control antibodies at 37°C for 1 h. Approximately 1.5 × 10^4^ per well of HEK293T-hACE2 cells were then added in duplicate to the above virus–antibody mixture. Approximately 48 h later, the half-maximal inhibitory concentration of each nanobodies and antibodies (IC50) was determined by luciferase activity using GraphPad Prism 8.3 (GraphPad Software).

### Nanobody neutralization against authentic SARS-CoV-2 and SARS-CoV-1

The focus reduction neutralization test (FRNT) was performed in a certified Biosafety level 3 laboratory. Neutralization assays against authentic SARS-CoV-2 were conducted using clinical isolate previously obtained from patients in Hong Kong, including D614G (MT835143), Delta (hCoV-19/Hong Kong/HKU-210804-001/2021; GISAID: EPI_ISL_3221329), Omicron BA.1 (hCoV-19/Hong Kong/HKU-691/2021 (HKU691); GISAID:EPI_ISL_7138045), Omicron BA.2 (GISAID: EPI_ISL_9845731), Omicron BA.5.2 (GISAID: EPL_ISL_13777658), Omicron BQ.1.1 (GISAID: EPL_ISL_16342297), Omicron XBB.1 (GISAID: EPI_ISL_15602393), Omicron XBB.1.5 (GISAID: EPI_ISL_17205250), EG.5.1 (GISAID: EPI_ISL_18461518), and SARS-CoV-1 (HKU-39849, GenBank: AY278491.2). Tnb04 was serially diluted and mixed with 50 μl of each of the authentic SARS-CoV-2 (MOI: 0.05) in 96-well plates and incubated for 1 hour at 37°C. Mixtures were then transferred to 96-well plates pre-seeded with 2 × 10^4^/well Vero-E6 TMPRSS2 cells and incubated at 37°C for 24 h. The culture medium was then removed, and the plates were fixed with a 4% paraformaldehyde solution for 30 min. Cells were further permeabilized with 0.2% Triton X-100 and incubated with cross-reactive rabbit sera anti-SARS-CoV-2-N (1:5000) for 1 hour at room temperate before adding an Alexa Fluor 488 goat anti-rabbit IgG (H + L) cross-adsorbed secondary antibody (1:1000 Life Technologies). The fluorescence density of SARS-CoV-2 infected cells were scanned using a Sapphire Biomolecular Imager (Azure Biosystems) and the neutralization effects (IC50) were then quantified based on the 50% reduction in fluorescence using Fiji software (ImageJ).

### Nanobody activity against cell-cell fusion

Inhibitory activity of nanobodies on spike-mediated cell-cell fusion was measured by a dual-split-protein (DSP)-based assay as previously described [45]. DSP involved a pair of chimeric reporter protein composed of split *Renilla* luciferase (RL) and split GFP. The spilt RL and GFP become self-reassociated and recover their activity upon cell-cell fusion. This assay provides quantitative analysis on membrane fusion mediated by viral envelops including spike of SARS-CoV-2. Briefly, the spike-expression cells (effector cells) were generated by co-transfecting HEK 293T cells with various spike-expressing plasmids and a nRL plasmid, and incubated 48 h at 37°C. The effector cells (2x10^4^/well) were digested and co-cultured with Huh7 targeted cells (5x10^4^/well) stably expressing cRL in 96-wells plates at 37°C in the presence or absence of a serial dilutions of testing antibodies. After incubation for about 6 hours, luciferase activity was measured with *Renilla*-Glo Luciferase Assay System (Promega) and IC50 values were calculated based on the 50% reduction compared to the no antibody controls. For fluorescence measurement, effector cells (1x10^5^/well) were co-cultured with targeted cells (5x10^4^/well) in 96-wells plates at 37°C overnight. The images of the cells were captured using the Opera Phenix Plus High-Content Screening System (PerkinElmer), and analyzed using the Harmony High-Content Imaging and Analysis Software 4.9.

### Crystallization and data collection

After a series of failure in obtaining crystal through RBD-nanobody binary complex, we decided to test out the ternary complex involving addition of antibody P2C-1F11, previously isolated from SARS-CoV-2 convalescent patient and shown to generate crystal with high propensity [46]. To this end, the Fab fragment of P2C-1F11 was mixed with RBD at a molar ratio of 1:1.2, incubated on ice for 1h, and purified by gel-filtration chromatography. The resultant binary complex was mixed with nanobody at a molar ratio of 1:1.5, incubated on ice for 1h, and further purified by gel-filtration chromatography. The purified ternary complex was concentrated to 10mg/mL in HBS buffer (10 mM HEPES, pH 7.2, 150 mM NaCl) for crystallization. The sitting drop vapor diffusion method was used by mixing 0.2 μL of ternary complex with 0.2 μL of reservoir solution and the screening at 18°C. We obtained the crystals of ternary complexes successfully in 0.2 M Ammonium phosphate dibasic, 20% w/v polyethylene glycol 3,350. Diffraction data were collected at the BL18U1 beamline of the Shanghai Synchrotron Research Facility (SSRF) and auto-processed with aquarium pipeline.

### Structural determination and refinement

The structure was determined by the molecular replacement method using PHASER (CCP4 Program Suite). Subsequent model building and refinement were performed using COOT and PHENIX. All structure figures were generated with ChimeraX and PyMOL (PMID: 28158668).

### Western Blot analysis for proteinase-K resistance core

SARS-CoV-2 spike (residues M1-Q1208), containing a foldon trimerization motif and a strep tag at C-terminal were expressed in the FreeStyle 293 F cells and purified by Strep-Tactin Sepharose followed by gel filtration chromatography. One μg of the spike was incubated with 2 μg of the ACE2 or 2 μg of nanobodies for 1 h on ice. Trypsin (final concentration of 5 μg/mL, ThermoFisher 27250018) was then added to this mixture and incubated for an additional 20 min at 37°C. Subsequently, the samples were subjected to proteinase-K (ThermoFisher AM2546) digestion with final concentration of 100 μg/mL and incubated 20 min at 37°C. 4×SDS-PAGE loading buffer was then added to all samples prior to boiling at 100°C. Samples were run on a 4–12% gradient Tris-MOPS-Gel (GenScript M00653) and transferred to polyvinylidene fluoride membranes. An anti-SARS-CoV- 2 S2 polyclonal antibody (SinoBiological T40590-T62, 1:2000 dilution) and an HRP-conjugated anti-rabbit secondary antibody (Promega W4011, 1:4000 dilution) were used for Western blotting. The image was developed by AI600 and the intensity of proteinase-K resistant core was estimated by ImageJ 1.53k.

### Antibody binding affinity measured by SPR

The binding affinity of nanobody-Fc to SARS-CoV-2 RBD and variant RBDs were analyzed by surface plasmon resonance (SPR) (Biacore 8K, GE Healthcare). Briefly, recombinant protein A (50ug/ml) (Sino Biological, China) were covalently immobilized to a CM5 sensor chip via amine groups in 10 mM sodium acetate buffer (pH 4.5) for 1000s. Diverse nanobody-Fc (20 μg/mL) in the running buffer (10mM PBS, pH 7.2 and 0.05% Tween 20) were injected and captured onto the sensor chip by recombinant protein A. Serial dilutions of prototype and variants RBDs of SARS-CoV-2 were flowed through the nanobody-Fc coated sensor chip system. The resulting data were analyzed by Single—cycle kinetics using capture-Evaluation method and the sensograms were fitted to a 1:1 binding model using Biacore Evaluation Software (GE Healthcare).

### Contact transmission of SARS-CoV-2 in hamsters

Hamsters were purchased from the Centre for Comparative Medicine Research (CCMR) of HKU. The animals were kept in Biosafety Level-2 housing and given access to standard pellet feed and water ad libitum following CCMR’s standard operational procedures (SOPs). The viral challenge experiments were then conducted in Biosafety Level-3 animal facility following SOPs strictly.

10 weeks-old male hamsters were used for *in vivo* evaluation of Tnb04-1 in preventing contact transmission of SARS-CoV-2 as described previously with slight modifications [49]. Briefly, 9 index hamsters were intranasally infected with 10^5^ PFU of SARS-CoV-2 XBB.1.5 (EPI_ISL_17205250) at 0 dpi. Naive hamsters (n = 6/group) were intranasally inoculated with 4.5 mg/kg Tnb04-1 at 2 hours or 8 hours before co-housing with index hamsters. Another 6 naïve hamsters intranasally inoculated with PBS at 2 hours before co-housing were included as control. At 2 dpi, each index hamster was co-housed with two naïve hamsters for 4 hours as a close contact. After co-housing, index hamsters were immediately sacrificed. Each naïve hamsters were then separated and housed in a new single cage. Naïve hamsters were sacrificed at day 2 after co-housing. Nasal turbinate and lung tissues were collected for viral load determination by quantitative SARS-CoV-2-specific genomic RdRp/Hel and subgenomic NP RT-qPCR assay as previously described[48, 51]. Infectious virus titration was performed by plaque assay. Specifically, serial 10-fold dilutions of each tissue homogenate were inoculated in a Vero-E6 TMPRSS2 cell monolayer in 12 well-plates. The plates were observed for cytopathic effects at day 3 post inoculation. Plaque-forming units (PFUs) were calculated by the number of plaques multiplied by the dilution factor and expressed as PFU/ml of tissue homogenate.

### Histopathological analysis and immunofluorescence staining

Lung and nasal turbinate sections were fixed in 4% formaldehyde solution and embedded in paraffin for the H&E and immunofluorescence staining. The whole tissue sections after H&E staining were scanned and analyzed using the Akoya Vectra Polaris Automated Quantitative Pathology Imaging System. The immunofluorescence staining was conducted for identification and localization of SARS-CoV-2 nucleocapsid protein (NP) using a rabbit anti-SARS-CoV-2-N antibody (1:5000) together with Alexa Fluor 488-conjugated goat anti-rabbit IgG antibody (1:1000 Life Technologies) as we previously described[48]. The images of the lung tissue section were captured using the Carl Zeiss LSM 780 confocal microscope and analyzed using the ZEN 3.3 software (Blue edition).

### Statical analysis

The technical and independent experiment replicates were indicated in the figure legends. Half-maximal and 90% inhibitory concentration (IC50 and IC90) of antibodies was calculated by the equation of four-parameter dose inhibition response using Graphpad Prism 8.0. All data are presented as mean ± SEM unless otherwise indicated. In animal experiments, a two-tailed unpaired Mann-Whitney test was used to assess statistical significance. Statistical calculations were performed in GraphPad Prism 8.0. Differences with p-values less than 0.05 were considered to be statistically significant (**p < 0.01).

## Supporting information

S1 Fig Alignment of amino acid sequences of the top 5 nanobodies. Sequences comparison of the top 5 nanobodies isolated here together with our previously isolated nanobody Tnb03 (3-2A2-4). The exact frame and CDR regions along the nanobody sequences are indicated. Dots represent the identical residues to Tnb03 (3-2A2-4).
(TIF)

S2 Fig Comparison of inhibition activity of Tnb04-1 against pseudoviruses, authentic viruses, and spike-mediated cell-cell fusion. The actual neutralizing and inhibition curve of Tnb04-1 against pseudoviruses, authentic viruses, and cell-cell fusion of eight SARS-CoV-2 variants.
(TIF)

S3 Fig Inhibition curves of the top 5 nanobodies against cell-cell fusion. The potency and breadth of the top 5 isolated nanobodies and control antibodies in inhibiting cell-cell fusion mediated by a panel of 20 spike variants.
(TIF)

S4 Fig Neutralizing curves of Tnb04-1 against SARS-CoV-2 KP.3 and KP.3.1.1. The actual neutralizing curve of Tnb04-1 against pseudoviruses bearing the full-length envelope of KP.3 and KP.3.1.1, from which the IC50 and IC90 are estimated. The results shown are representatives of two independent experiments and presented as mean ± SEM.
(TIF)

S5 Fig The binding angle of Tnb04-1 to the hydrophobic pocket of RBD relative to control antibodies. **a.** Tnb04-1 (green) approaches its epitope with 21.2° shift towards the outer face of RBD, relative to Tnb03 (3-2A2-4) (gray). **b.** Zoom in view of Tnb04-1’s CDR3 residues F102 and F103 (green) penetrating into a hydrophobic pocket of RBD, compared with that of Tnb03 (3-2A2-4) (gray). **c.** Binding to the hydrophobic pocket through different yet related CDR3 residues among Tnb04-1 (F102 and F103), 47D11 (W102 and F103), Beta-49 (W102), and Beta-50 (W102).
(TIF)

S6 Fig Images of infected lungs and NT from all hamsters. Images of infected **a.** lungs and **b.** NT from all hamsters as determined by anti-NP immunofluorescence (IF) staining. Cell nuclei were counterstained with DAPI (blue). Images were captured using the Carl Zeiss LSM 900 confocal microscope and analyzed using the ZEN 3.3 software (Blue edition).
(TIF)

S1 Data Source Data for Figs 1–7, S2, and S4. (XLSX)
