# Supplementary figures and images for: Super broad and protective nanobodies against Sarbecoviruses including SARS-CoV-1 and the divergent SARS-CoV-2 subvariant KP.3.1.1
Source: PLoS Pathog. 2024 Nov 11;20(11):e1012625. doi: 10.1371/journal.ppat.1012625 (PMC11554226; doi:10.1371/journal.ppat.1012625)

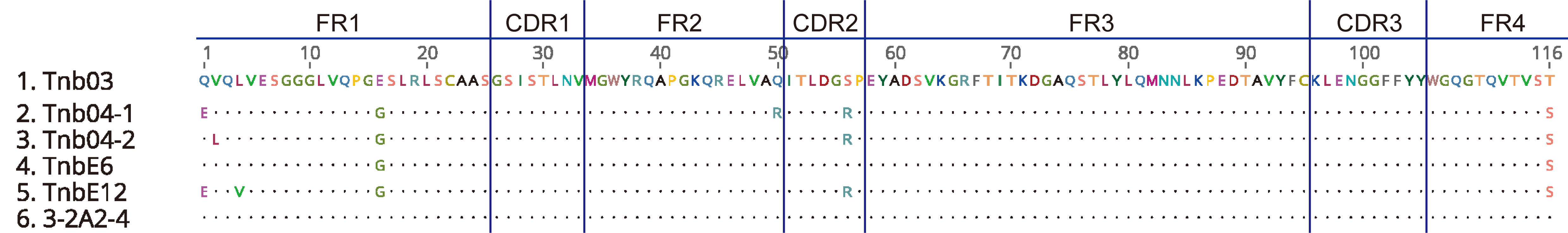

Supplement: S1 Fig — Sequences comparison of the top 5 nanobodies isolated here together with our previously isolated nanobody Tnb03 (3-2A2-4). The exact frame and CDR regions along the nanobody sequences are indicated. Dots represent the identical residues to Tnb03 (3-2A2-4). (TIF) [file ppat.1012625.s001.tif]

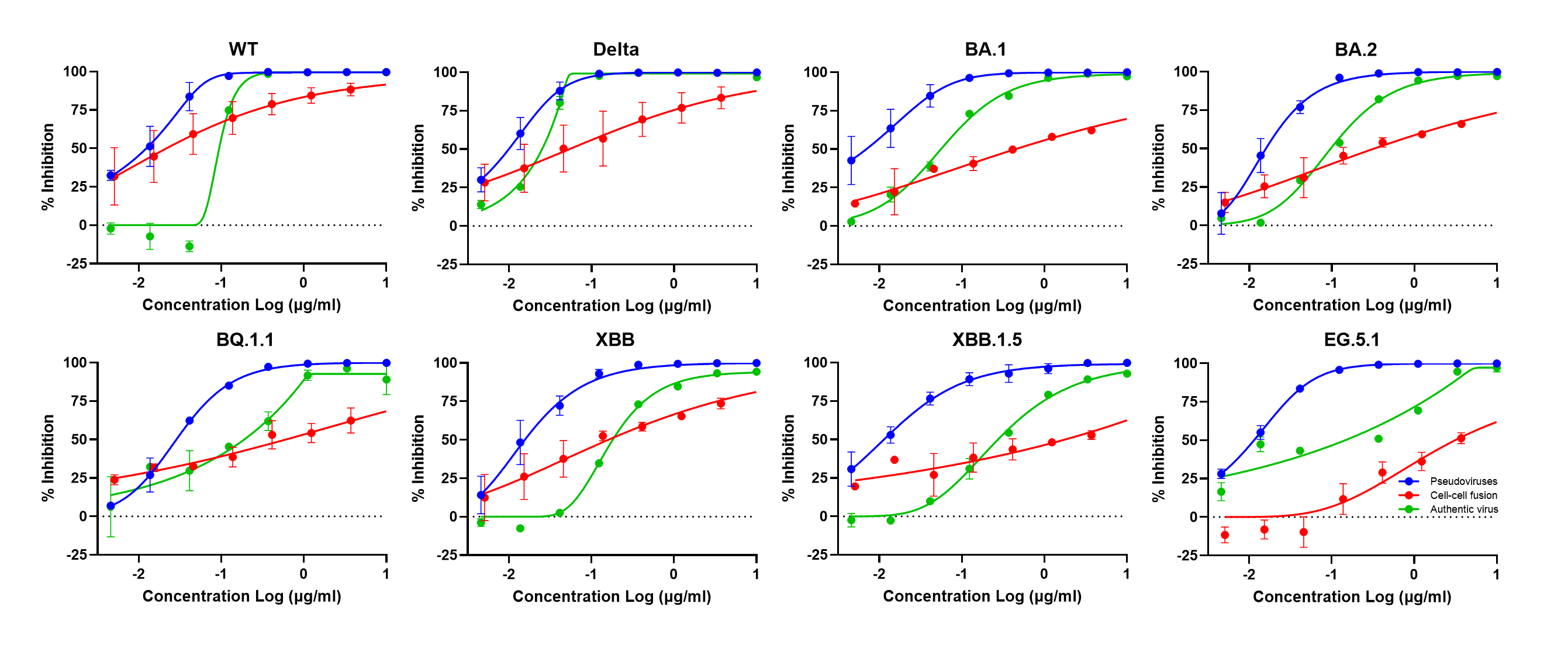

Supplement: S2 Fig — The actual neutralizing and inhibition curve of Tnb04-1 against pseudoviruses, authentic viruses, and cell-cell fusion of eight SARS-CoV-2 variants. (TIF) [file ppat.1012625.s002.tif]

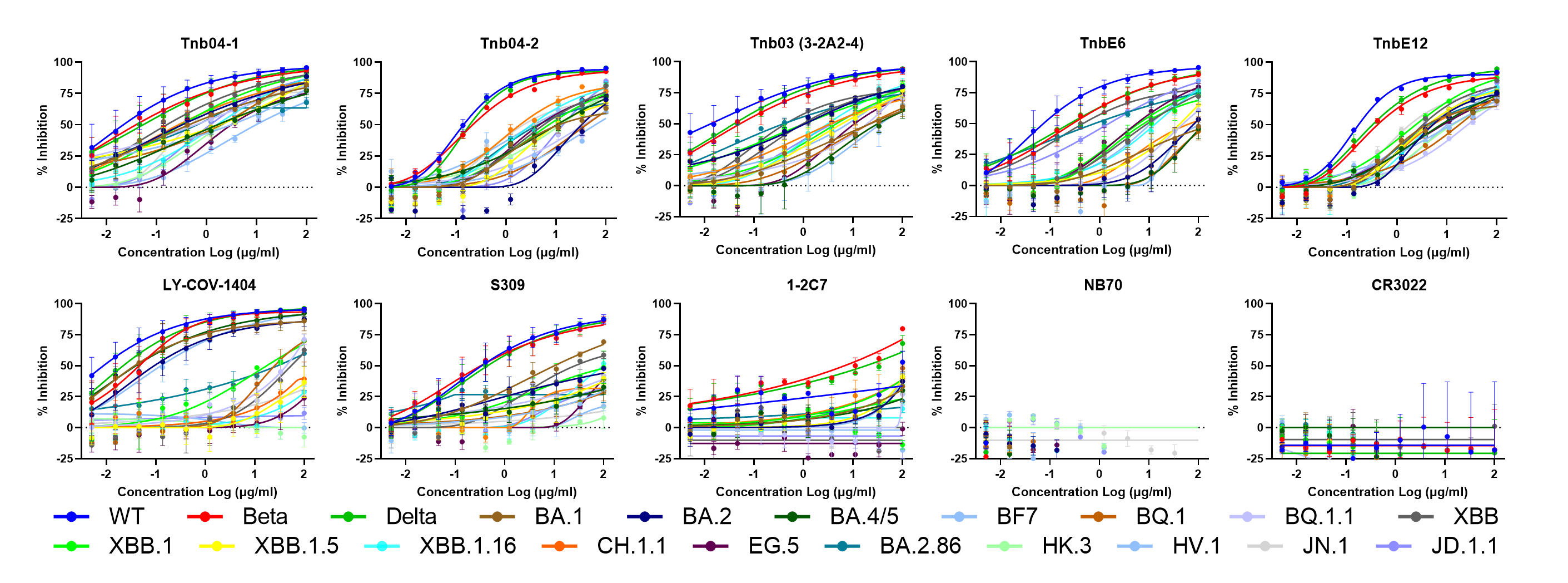

Supplement: S3 Fig — The potency and breadth of the top 5 isolated nanobodies and control antibodies in inhibiting cell-cell fusion mediated by a panel of 20 spike variants. (TIF) [file ppat.1012625.s003.tif]

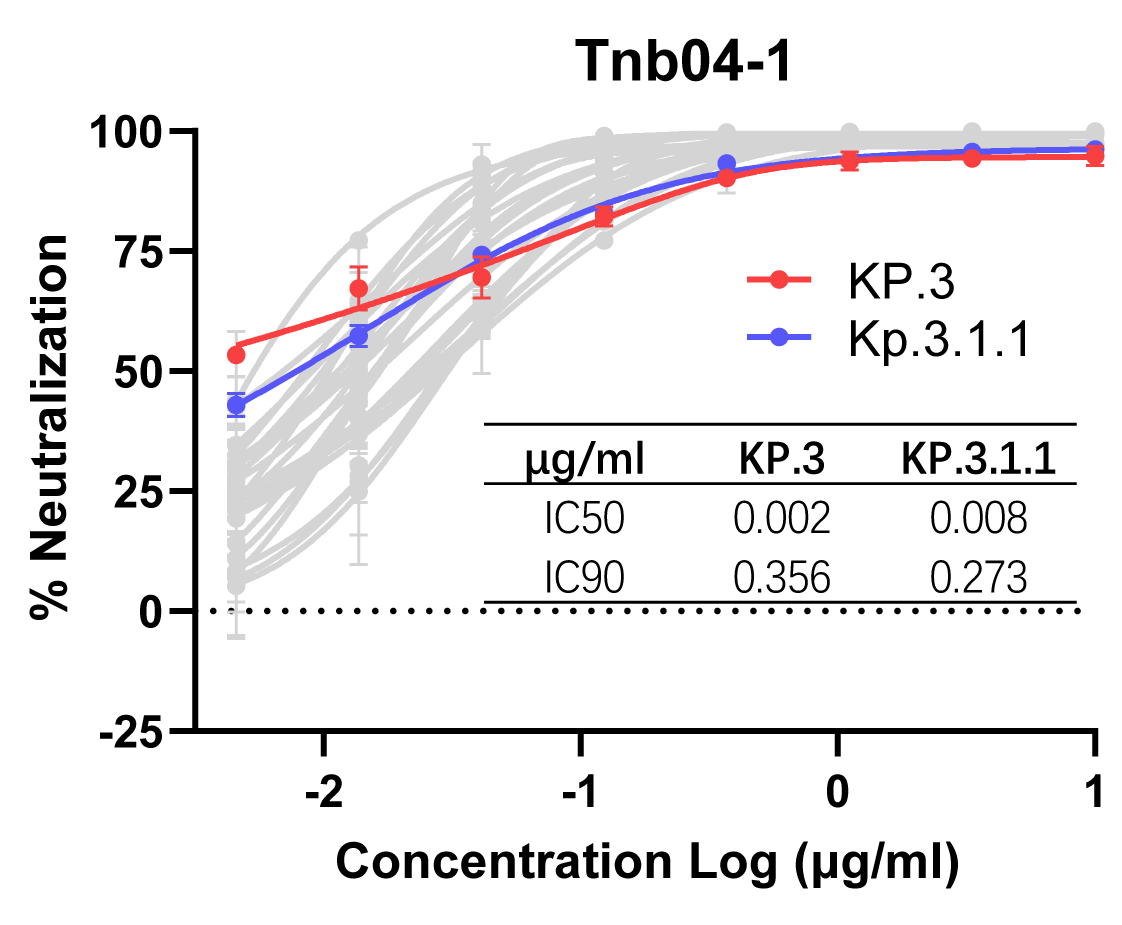

Supplement: S4 Fig — The actual neutralizing curve of Tnb04-1 against pseudoviruses bearing the full-length envelope of KP.3 and KP.3.1.1, from which the IC50 and IC90 are estimated. The results shown are representatives of two independent experiments and presented as mean ± SEM. (TIF) [file ppat.1012625.s004.tif]

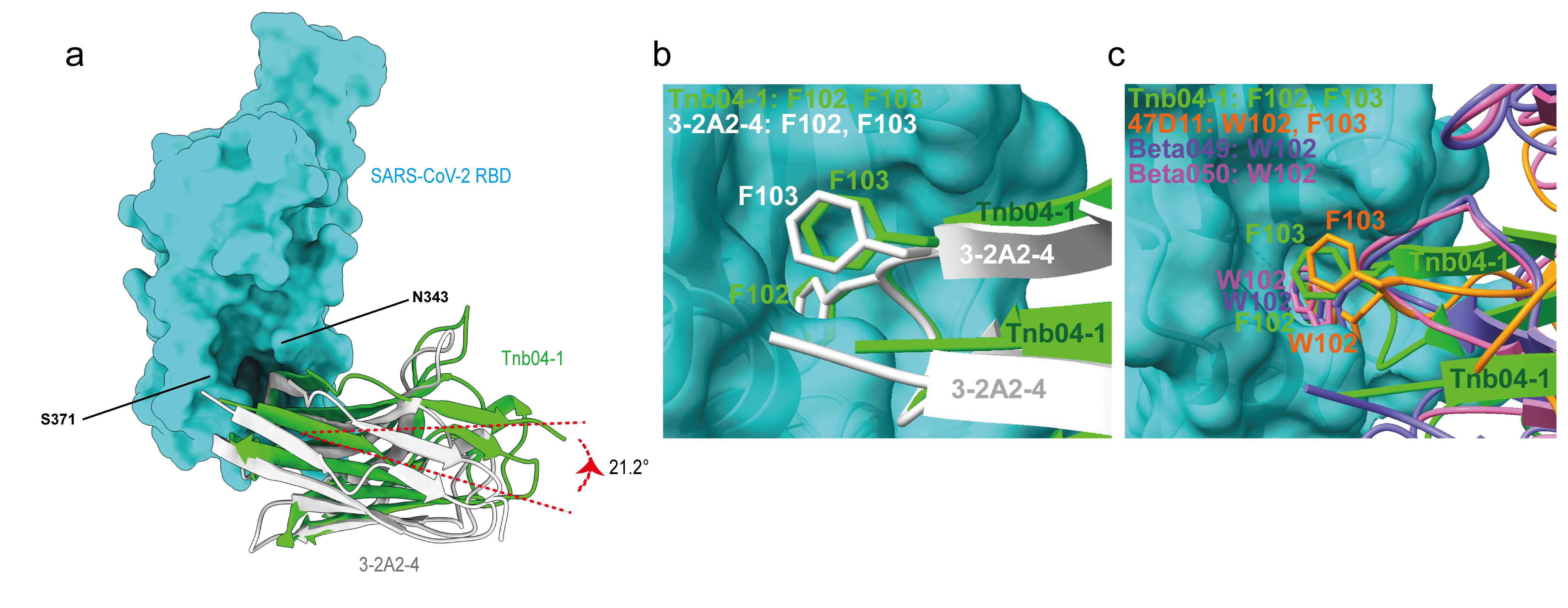

Supplement: S5 Fig — a. Tnb04-1 (green) approaches its epitope with 21.2° shift towards the outer face of RBD, relative to Tnb03 (3-2A2-4) (gray). b. Zoom in view of Tnb04-1’s CDR3 residues F102 and F103 (green) penetrating into a hydrophobic pocket of RBD, compared with that of Tnb03 (3-2A2-4) (gray). c. Binding to the hydrophobic pocket through different yet related CDR3 residues among Tnb04-1 (F102 and F103), 47D11 (W102 and F103), Beta-49 (W102), and Beta-50 (W102). (TIF) [file ppat.1012625.s005.tif]

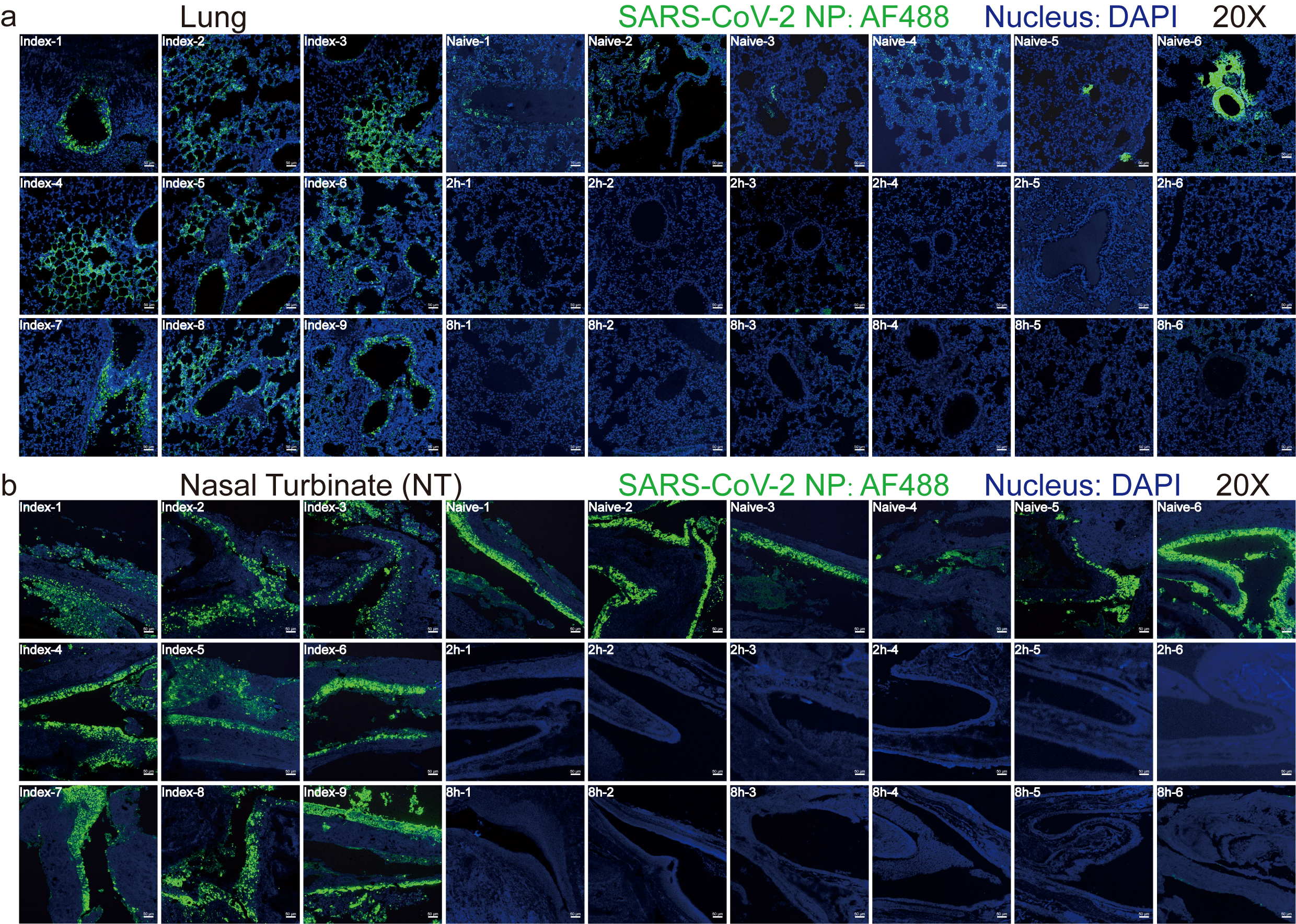

Supplement: S6 Fig — Images of infected a. lungs and b. NT from all hamsters as determined by anti-NP immunofluorescence (IF) staining. Cell nuclei were counterstained with DAPI (blue). Images were captured using the Carl Zeiss LSM 900 confocal microscope and analyzed using the ZEN 3.3 software (Blue edition). (TIF) [file ppat.1012625.s006.tif]
